# Mutation in *Drosophila concentrative nucleoside transporter 1* alters spermatid maturation and mating behavior

**DOI:** 10.3389/fcell.2022.945572

**Published:** 2022-08-23

**Authors:** Houda Ouns Maaroufi, Lucie Pauchova, Yu-Hsien Lin, Bulah Chia-Hsiang Wu, Lenka Rouhova, Lucie Kucerova, Ligia Cota Vieira, Marek Renner, Hana Sehadova, Miluse Hradilova, Michal Zurovec

**Affiliations:** ^1^ Biology Centre of the Czech Academy of Sciences, Institute of Entomology, Ceske Budejovice, Czechia; ^2^ Faculty of Science, University of South Bohemia, Ceske Budejovice, Czechia; ^3^ Institute of Molecular Genetics, Czech Academy of Sciences, Prague, Czechia

**Keywords:** cnt1, gamete, spermatogenesis, testis, adenosine, copulation, mitochondria, male fertility

## Abstract

Concentrative nucleoside transporters (Cnts) are unidirectional carriers that mediate the energy-costly influx of nucleosides driven by the transmembrane sodium gradient. Cnts are transmembrane proteins that share a common structural organization and are found in all phyla. Although there have been studies on Cnts from a biochemical perspective, no deep research has examined their role at the organismal level. Here, we investigated the role of the *Drosophila melanogaster cnt1* gene, which is specifically expressed in the testes. We used the CRISPR/Cas9 system to generate a mutation in the *cnt1* gene. The *cnt1* mutants exhibited defects in the duration of copulation and spermatid maturation, which significantly impaired male fertility. The most striking effect of the *cnt1* mutation in spermatid maturation was an abnormal structure of the sperm tail, in which the formation of major and minor mitochondrial derivatives was disrupted. Our results demonstrate the importance of *cnt1* in male fertility and suggest that the observed defects in mating behavior and spermatogenesis are due to alterations in nucleoside transport and associated metabolic pathways.

## Introduction

Male fertility in *Drosophila* depends on a number of traits, including appropriate mating behavior and proper progression of spermatogenesis (Baptissart et al., 2013; Lotti and Maggi, 2018). Mating is a complex process that is essential for reproductive success. The male pursues the female while vibrating his wings to produce a courtship song, tapping the female’s abdomen with his forelegs, touching the female’s genitals with his mouthparts, and curving his abdomen for copulation (Pavlou and Goodwin, 2013; Ziegler et al., 2013). The transfer of mature and healthy sperm to the female genitalia also depends on proper spermatogenesis, which involves the proliferation and differentiation of spermatogonial stem cells (Wakimoto et al., 2004; Yuan et al., 2019).

Spermatogenesis in *Drosophila* begins with stem cell proliferation, wherein the stem cell undergoes four mitotic divisions with incomplete cytokinesis, forming a cyst with 16 interconnected spermatogonia. This is followed by cell growth and meiosis to produce a syncytial group of 64 spherical haploid spermatids (Steinhauer, 2015). Subsequently, these spermatids undergo elongation and individualization (Tokuyasu, 1975; Noguchi and Miller, 2003), a process known as *Drosophila* spermiogenesis, in which the spermatids are remodeled into spermatozoa (Alzyoud et al., 2021). Spermatogonia develop in an enclosure formed by two cyst cells of somatic origin that undergo extensive morphogenesis and eventually differentiate into the head and tail cysts during spermatid elongation (Lindsley and Tokuyasu, 1980; White-Cooper, 2004). During this process, the round-shaped spermatid nuclei become long and thin (needle-shaped nuclei), accompanied by chromatin condensation. The fully elongated spermatids begin the individualization process at the basal end of the testis in the region of the terminal epithelium (TE), where actin-based individualization complexes (ICs) form around the nuclei then migrate from the heads to the tip of the tails. As ICs move along the elongated spermatid, unnecessary organelles and additional cytoplasm are stripped off and form a cystic bulge (CB). Once the cyst detaches from the tail, it forms a rounder waste bag (WB), which is eventually degraded, leaving each mature spermatozoon enclosed in its own tight membrane (Bader et al., 2010; Fabian and Brill, 2012). At the end of spermatogenesis, each mature sperm group coils in the TE region and is released in the testis lumen before migrating from the TE region to the seminal vesicle (SV), where it is prepared for ejaculation (Fabian and Brill, 2012; Demarco et al., 2014).

More than 2000 mutations affecting male fertility have been discovered in *Drosophila* (Wakimoto et al., 2004), but most of these mutations are not well characterized. Moreover, a number of genes with specific expression in the testis have not been well studied (Wakimoto et al., 2004; Zhao et al., 2010; Vedelek et al., 2018; Witt et al., 2019). Previous studies have shown that some of the mutations that affect mating behavior are connected to *Drosophila* morphology (*white* and *yellow* (Zhang and Odenwald, 1995)), learning and memory (*dunce* and *amnesiac* (Siegel and Hall, 1979)), sex determination (*fruitless* (*fru*) (Baker et al., 2001; Rideout et al., 2007)), or copulation (*Phosphodiesterase 1c* (*pde1*) (Morton et al., 2010)). Most of the spermatogenesis-related mutations affect germline cells (e.g., *zn finger homeodomain 1* and *delta* (Leatherman and Dinardo, 2008; Ng et al., 2019)), spermatocytes (e.g., *cannonball* (Chen et al., 2005)) and spermatids (e.g., *driceless* and *dynein intermediate chain at 61B* (*dic61B*)) (Huh et al., 2004; Fatima, 2011)). Other mutations may regulate the number (e.g., *headcase* (Resende et al., 2013)), morphology (e.g., *salto* (Augière et al., 2019)), or motility (e.g., *hemingway* (Soulavie et al., 2014)) at different cell stages.

We studied a gene encoding the *Drosophila concentrative nucleoside transporter 1* (*cnt1*), which is expressed in a testis-specific manner (Larkin et al., 2021). Cnt1 is a unidirectional carrier that mediates the energy-costly influx of nucleosides driven by the transmembrane sodium gradient (Gray et al., 2004; Chintapalli et al., 2007; Larkin et al., 2021). Nucleoside transporters and the enzymatic activities of adenosine metabolism affect many physiological processes by influencing the adenosine levels as a signaling molecule, especially the role of the adenosine signal terminator at the respective receptor (Knight et al., 2010). Cnts are found in most organisms, including mammals and insects, and are fairly conserved among phyla. For example, Cnts in humans and *Drosophila* have 24–33% sequence similarity (Machado et al., 2007). The involvement of *Drosophila* Cnt1 and Cnt2 in adenosine transport was examined in a previous study that showed that knocking down their expression rescues Cl.8 + tissue culture cells from the cytotoxic effect of a high concentration of extracellular adenosine (Fleischmannova et al., 2012). The Cnt protein structure is characterized by 13 transmembrane domains (Young et al., 2013; Dos Santos-Rodrigues et al., 2015), and they seem to have limited tissue distribution within organisms. For example, in mammals, Cnts have been detected in specialized cells, particularly intestinal and hepatic epithelial cells and testis (Kato et al., 2005; Masino and Boison, 2013). Cnts have been identified in rat epididymis and shown to play a role in sperm maturation (Leung et al., 2001; Klein et al., 2017). In addition, in a recent study, the deficiency of human Cnt1 has been associated with a newly discovered inborn error of pyrimidine metabolism and organ failure in an infant (Pérez-Torras et al., 2019).

In the present study, we identified the *cnt1* gene as an important component in mediating the crosstalk between fertility and systemic metabolism in *Drosophila*. We showed that Cnt1 is highly expressed in testes. To determine the role of *cnt1* in *Drosophila*, we created a mutation in this gene using the CRISPR/Cas9 system and named the obtained allele *cnt1*{*fertility defect*} (*cnt1* ^*FD*^). The mutation in *cnt1* caused a defect in mating behavior (copulation duration) and spermatid maturation that significantly impaired male fertility. The prominent effect of the *cnt1* ^*FD*^ mutation in spermatid maturation involved an abnormal structure of the spermatid tail and low sperm count.

## Materials and methods

### Fly stocks

Fly strains were reared on a standard cornmeal medium at 25°C with 12/12 h light/dark cycle and 60% relative humidity. *Cnt1* ^*FD*^ mutant and *cnt1::GFP* were used and generated as described below. *W* ^*1118*^ was used as a wild-type control. Flies containing *protA::GFP* were provided by Dr. R. Renkawitz-Pohl and Dr. Ch. Rathke (Jayaramaiah Raja and Renkawitz-Pohl, 2005), while the *dj::GFP* (BL 5417, USA) and *Df*(*2R*)*BSC271* (BL 23167, USA) were obtained from Bloomington (BL5417, USA).

### Generation of the *cnt1* gene mutation

The mutation in the *Drosophila cnt1* gene was generated through the CRISPR/cas9 gene editing approach (Kondo and Ueda, 2013). Two guide RNA (gRNA) [GGC​AAA​GCG​AAT​CAC​CTA​TCT​GG and GTG​CCG​CAA​TGA​CGC​ATA​CAA​GG] were used to create a deletion. The first was located in the first coding exon and the second in the third coding exon. The double-strand break was corrected by non-homologous end-joining, which led to an open reading frame deletion of 477 bp and substitution of one amino acid at the break site (the entire *cnt1* deletion comprises 752 bp, including coding exon 1, complete exon 2, part of coding exon 3, and two complete introns.). The *cnt1* ^*FD*^ mutants were generated by injecting the described construct into the embryos of nanos-cas9 expressing flies and examined through polymerase chain reaction (PCR). The flies were isogenized to the background of *w* ^*1118*^ flies*.* Both male and female flies were homozygous viable for this deletion. We confirmed the mutation in *cnt1* (*cnt1* ^*FD*^) with the primers at the deletion site (Supplementary Table S1).

### Generation of *cnt1*-tagged GFP

We generated flies containing a genomic sequence of *cnt1* tagged with *GFP* (*cnt1::GFP*) (Ejsmont et al., 2011). We cloned the GFP sequence into a fosmid vector containing the *cnt1* gene as a pre-tagging vector (Ejsmont et al., 2011; Sarov et al., 2016). The tagged clone was later injected into *Drosophila* embryos using GenetiVision services (Groth et al., 2004). The mutation in the flies was verified via PCR according to manufacturer protocol (Thermo Scientific Phusion High-Fidelity PCR Master Mix). The transgenic flies, *cnt1::GFP*, were used to trace the expression pattern of c*nt1* through the integrated *GFP* and used as a rescuers by combining it with the *cnt1* ^*FD*^ mutant fly.

### Real-time quantitative reverse transcription-PCR

The RT-qPCR was performed as previously described (Lin et al., 2019). Total RNA was extracted from adult flies using TRIzol reagent (#1559026, Invitrogen, USA). Thereafter, the isolated RNAs were treated with DNase using the Promega RQ1 RNase-free DNase kit to prevent genomic DNA contamination. The DNase-treated RNAs were used for cDNA synthesis from 2 μg total RNA using a RevertAid H Minus First Strand cDNA Synthesis Kit (Thermo Fisher Scientific, Vilnius, Lithuania). The cDNA was amplified by RT-qPCR using the 5 × Hot FirePol EvaGreen qPCR Mix Plus with ROX (Solis BioDyne, Tartu, Estonia) and Rotor Gene Q Instrument (QIAGEN, Hilden, Germany). The primers used for *cnt1* quantification are presented in Supplementary Table S1. *αTub84B* (FBgn0003884) and *act5C* (FBgn0000042) were used as reference housekeeping genes. The expression level was calculated using the 2^–ΔΔCt^ method after normalizing the ct values of the target genes to the reference gene.

### Fertility test

Virgin male and female flies were collected and separated for 3 days. Then, each of the adult male flies was placed in a vial along with 10 *w* ^*1118*^ virgin female flies (3 days old) at 25°C. After 24 and 48 h, the females were placed in individual vials and examined for the appearance of offspring. The number of fertilized females per male was recorded.

### Copulation assay

For the copulation length assay, virgin male and female flies were collected and separated for 3 days. Thereafter, the flies were sedated, placed in individual vials, and left to recover for 1 h. Then, a single male and a female were placed in a common vial, and video recording was undertaken.

For the copulation frequency assay, virgin male and female flies were collected and separated for 3 days. Then, a single male and 10 female flies were placed in a vial. Afterwards, the number of copulations was recorded.

### Histology: Immunohistochemical staining

Immunohistochemical staining was performed as described previously with a few modifications (Lin et al., 2021). *Drosophila* adult testes were dissected in 1 × phosphate buffer saline (PBS) and fixed in 4% paraformaldehyde for 20 min. After washing with PBST (0.3% Triton in 1× PBS) three times, the testes were incubated in a blocking buffer (5% goat serum in 0.1% PBST) for 1 h. Next, the blocking buffer was replaced by primary antibodies mixed in PAT (1% BSA, 0.1% Triton and 0.01% sodium azide in 1× PBS) and the incubation lasted for overnight. Then, the testes were washed three times with 0.1% PBST and incubated in secondary antibodies mixed in 0.1% PBST for 1 h. After washing with 0.1% PBST three times, the tissues were stained with DAPI for 10 min and washed three times before mounting with Fluoromount-G. DAPI (#MBD0015, Sigma-Aldrich, Germany), DCP1 (#9578S, Cell signaling, USA).

### Image analysis

Images of the stained testes were taken using a laser scanning confocal microscope FluoView™ FV1000 (Olympus, Tokyo, Japan). To quantify the canoe-stage spermatids, sperms, and waste bags, the area of the TE region was obtained using ImageJ software (Schneider et al., 2012).

### Ultrathin section of *Drosophila* testis specimens

The testes dissected in PBS were fixed in 2.5% glutaraldehyde for at least 4 h at room temperature (RT) or overnight at 4°C. The specimens were placed in a wash solution containing PBS with 4% glucose (three times for 15 min at RT) and then treated with a 1:1 mixture of PBS and 4% OsO4 solution (for 2 h at RT). After application of the wash solution (three times for 15 min at RT), the tissues were dehydrated in an acetone series (30, 50, 70, 80, 90, 95, and 100% for 15 min each). The dehydrated samples were embedded in Epon resin by gradually increasing the volume ratio of resin to acetone (1:2, 1:1, and 2:1 for 1 h each at RT). The specimens were left in the resin for 24 h (RT), and then the resin was polymerized at 62°C for 48 h. The ultrathin sections were cut with a diamond knife and stretched with chloroform. The sections were placed on copper meshes and contrasted with uranyl acetate and lead citrate, after which they were coated with carbon. The samples were imaged under the JEOL JSM-7401F transmission electron microscope (JEOL, Akishima, Japan).

### Statistical analysis

Graphs were produced in GraphPad Prism 5 software. Statistical analysis of the data was executed using Statistica software (StarSoft CR) and Microsoft Excel. Significance was established using parametric tests: Student’s t-test (N.S., not significant, *p < 0.05, **p < 0.01, ***p < 0.001) and one-way ANOVA (combined with Tukey post-hoc test), or non-parametric test: Kruskal–Wallis (combined with comparisons of mean ranks of all pairs of groups post-hoc test). Statistical analysis of the RT-qPCR was done using log2 value (Supplementary Table S2).

### RNAseq

The *Drosophila* testes were dissected and frozen in liquid nitrogen. Biological triplicates were prepared, and 40 individuals were dissected for each replicate. TRIzol (#1559026 Invitrogen, USA) was applied to each sample, and RNA was isolated according to manufacturer protocol. The isolated RNA was further purified and treated by cDNAse using a NucleoSpin RNA kit (#740955.250 Macherey-Nagel, Germany). The quality of total RNA was controlled using an RNA 6000 Nano Kit on an Agilent Bioanalyzer 2,100 and the quantity using an RNA BR Assay Kit on a Qubit 2.0 Fluorometer (Life Technologies, USA). The sequencing library was prepared from a 1,000 ng input of high-quality (RIN >7) total RNA using a KAPA mRNA HyperPrep Kit for Illumina Platforms (Roche, KK8580, Switzerland) according to manufacturer protocol, with fragmentation conditions set at 94°C and 7 min. The final library PCR amplification was set at nine cycles. Library length profiles and concentrations were monitored using an Agilent Bioanalyzer (High-Sensitivity DNA chip) and a Qubit 2.0 Fluorometer (dsDNA HS Assay Kit), respectively. A pool of libraries in equimolar ratio was generated and sequenced in single-end mode on the NextSeq 500 Illumina platform using the NextSeq 500/550 High Output Kit v2.5 (75 Cycles) with a loading molarity of 1,8 p.m. including 1% PhiX control. Data analysis was performed using Galaxy online software and its associated tutorial (Batut et al., 2018). Our SRA database has been released on NCBI under the accession: PRJNA838856.

### Phylogeny tree

Representative protein sequences of Cnt homologs, namely Sodium/Nucleoside Cotransporters or Solute Carrier Family 28 Members-3, covering the phylogeny of the genus *Drosophila* and the phylogeny of insects were identified using the Basic Local Alignment Search Tool (BLAST) in the GenBank database (Clark et al., 2016). Amino acid sequences were then aligned using the software MUSCLE (Edgar, 2004). Smart Model Selection, an online software (Lefort et al., 2017), was used to pick the best model according to the lowest Bayesian Information Criterion (BIC) score. Phylogenetic analyses were conducted through the Maximum Likelihood approach in PhyML 3.0 (Guindon et al., 2010). For each branch, statistical support was calculated as aBayes values. Trees were finalized with the aid of MEGA version X (Kumar et al., 2018).

## Results

### Phylogeny of Cnts in insects and drosophilidae family

**FIGURE 1:**
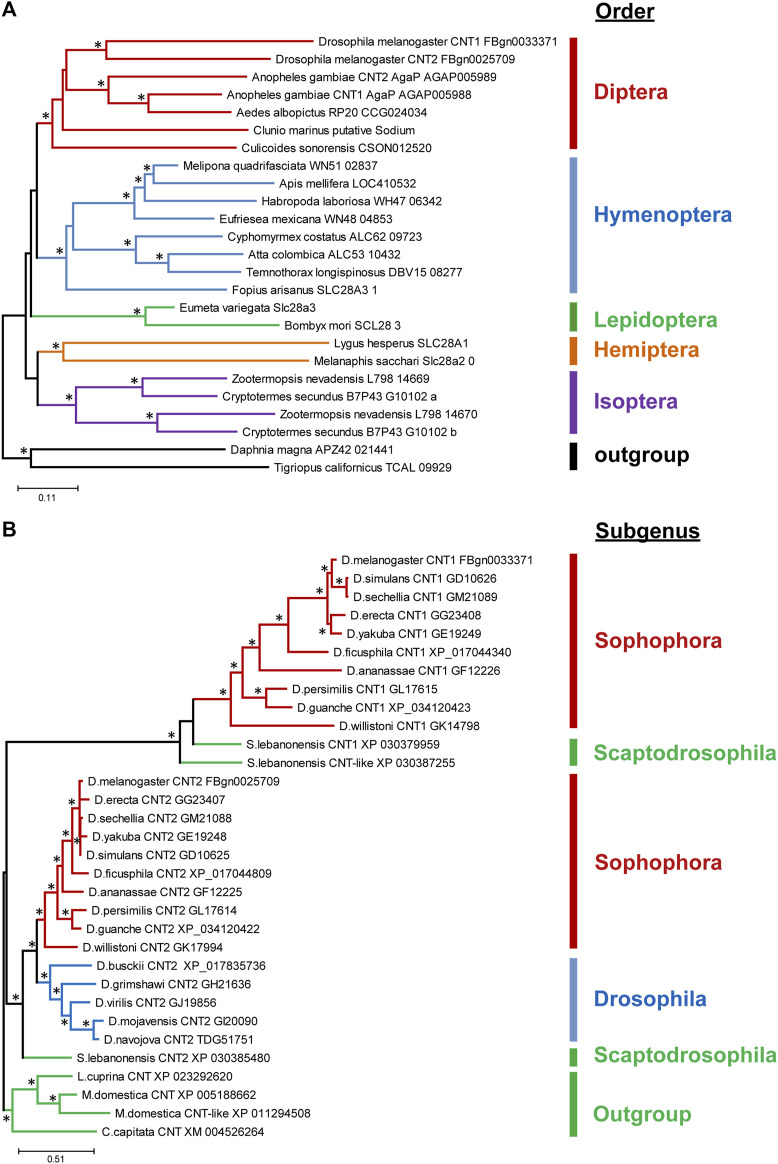
Distribution of Cnts in insects and family drosophilidae. **(A)** Simplified phylogenetic tree of Cnts among insects. Each color represents a particular order of insects. **(B)** Simplified phylogenetic tree of Cnt in *Drosophila*. Each color represents a subgenus of *Drosophila*. Branches with statistical support (aBayes values) greater than 70 are marked with asterisks.

The *cnt* genes are present in most living organisms, including eubacteria, suggesting that they are evolutionarily ancient (Young et al., 2013). Different taxonomic groups may differ in the number of *cnt* isoforms. For example, insects have one to three *cnt* isoforms, whereas mammals have three different *cnt* genes (Molina-Arcas and Pastor-Anglada, 2013; Young et al., 2013; Young, 2016). Moreover, among insects, Isoptera and Diptera seem to be the only insect orders where duplication events occur (Figure 1A). The duplication within the Isoptera appears to be a single origin event, whereas in Diptera, there seem to be multiple duplications and gene loss events. A closer look at the distribution of *cnts* within the genus *Drosophila* reveals a recent duplication of *cnt* in the common ancestor of *Drosophila* and *Scaptodrosophila*, with a secondary loss of *cnt1* in the subgenus *Drosophila* (Figure 1B). Based on the alignments among insects, the Cnts differ mainly by the first 20 amino acids, while the rest of the sequences are well conserved (Supplementary Figure S1).

The presence of Cnts in all species indicates that they perform important functions in organisms. The presence of multiple isoforms in some *Drosophila* species suggests that they may also be required in a tissue-specific manner.

### *cnt1* is associated with male fertility

**FIGURE 2:**
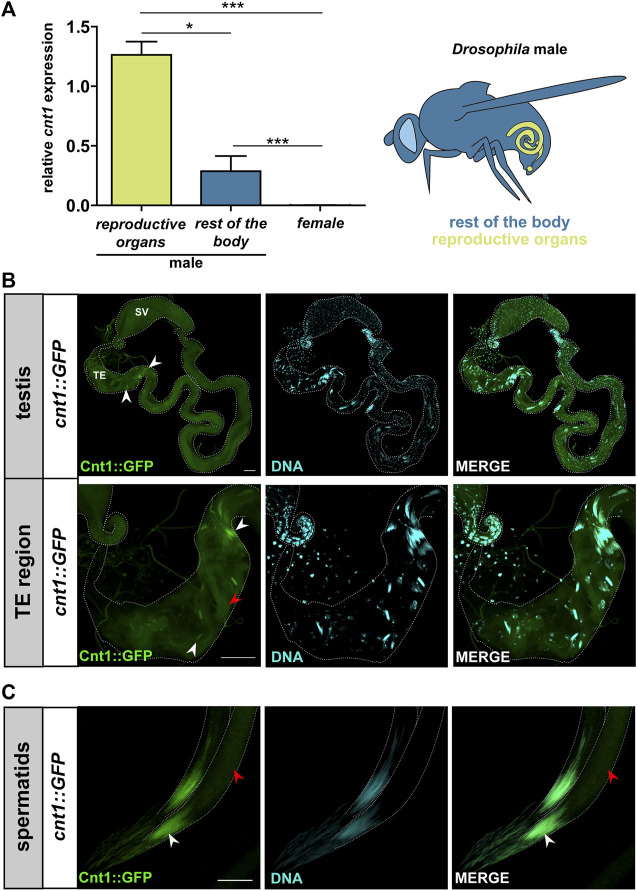
Cnt1 expression in the head and tail of spermatids. **(A)** The results of RT-qPCR analysis show a relative expression of *cnt1* in the reproductive organs and the rest of the body of the *w* ^*1118*^ male and female flies (see the illustration). Expressions were normalized to *αTub84B* and *act5C* transcripts (ΔΔCT). Significance was analyzed by one-way analysis of variance (ANOVA) and labeled as follows: *p < 0.05, ***p < 0.001; n = 5. Error bars are given as mean ± SEM. **(B)** Cnt1::GFP localization in *Drosophila* testes and its TE region. Scale bar: 40 µm. **(C)** A closer look at the localization of Cnt1::GFP in spermatid heads (needle-stage spermatids). Scale bar: 10 μm. Cnt1::GFP staining in green and DNA staining (DAPI) in cyan. Head of spermatids (white arrowhead), tail of spermatids (red arrowhead), terminal epithelium (TE), seminal vesicle (SV).

According to FlyBase, *cnt1* is highly expressed in the male testes (Larkin et al., 2021). To confirm this expression profile, we performed RT-qPCR using *cnt1* gene-specific primers on the extracted reproductive organs and the rest of the body of *w* ^*1118*^ male flies and females (Supplementary Table S1). Data analysis showed a high expression of *cnt1* in males and especially in the testes rather than the rest of the body organs in males (Figure 2A). We also used the fosmid *FlyFos* transgene (Ejsmont et al., 2009) and analyzed the expression of *GFP*-tag (Sarov et al., 2016) (*cnt1::GFP*) in *w* ^*1118*^ background. A well-defined GFP signal was found in the TE region of the testis in flies carrying the *cnt1::GFP* transgene but not in the control *w* ^*1118*^ (Figure 2B and Supplementary Figure S2). This signal was located in the head of the canoe- and needle stage-spermatids and overlapped with the DNA staining. In addition, a weak GFP signal was detected in the spermatid tail (Figures 2B,C). Detailed analysis showed that Cnt1 expression begins during spermiogenesis when late canoe-stage spermatids start to form, and that the signal persists until the formation of needle-stage spermatids (Figure 2C). These results confirm the tissue specificity of Cnt1 expression.

**FIGURE 3:**
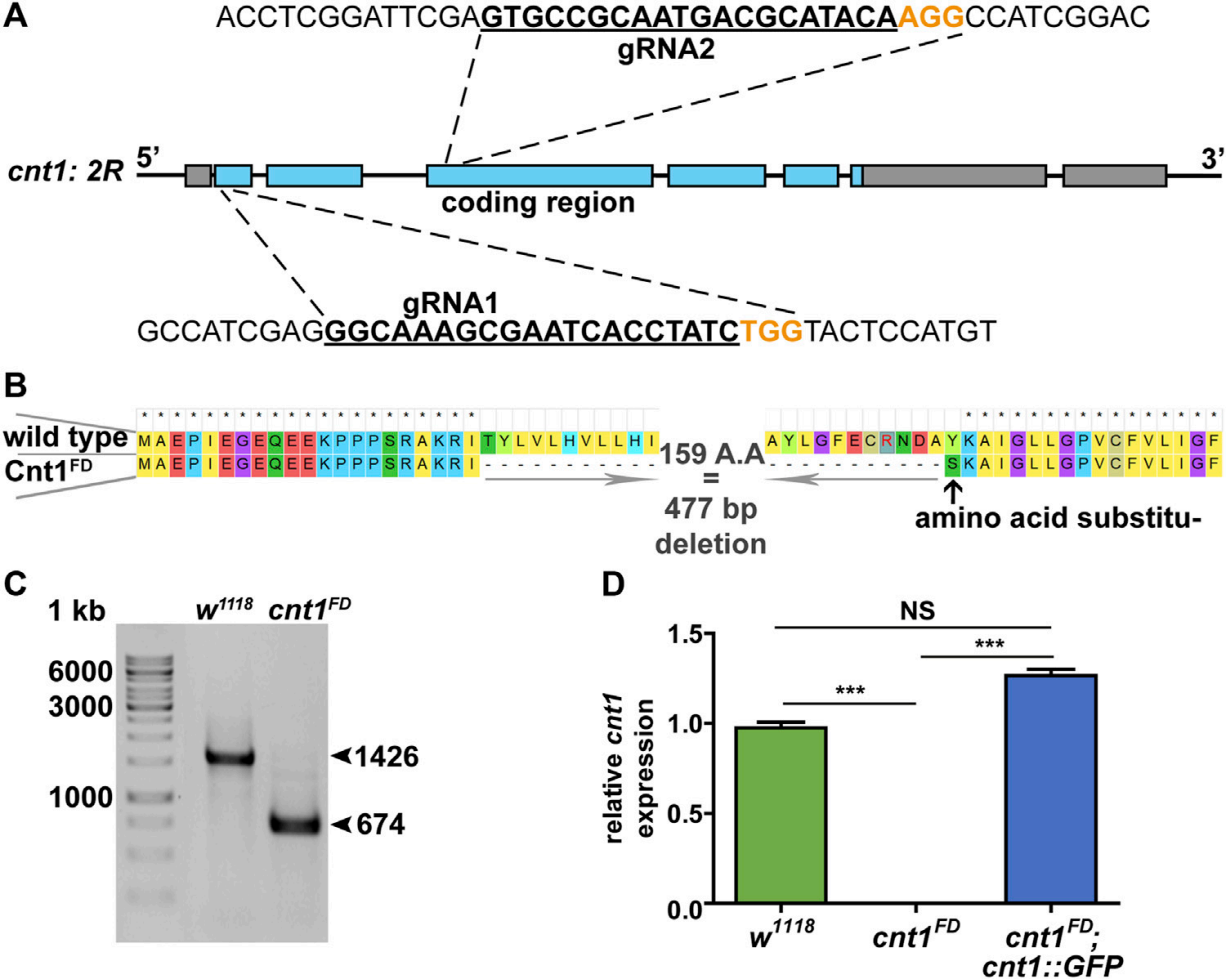
Generation of mutation in *Drosophila cnt1*. **(A)** Schematic representation of the *cnt1* mutation using two guide RNAs (gRNA); the target of the first gRNA is located in the first coding exon and the second in the third coding exon. **(B)** Protein sequence alignment of Cnt1 in control *w* ^*1118*^ and *cnt1* ^*FD*^. The mutation caused a deletion of 477 bp (159 A.A) in the coding region of *cnt1* gene (a total of 752 bp including introns) and a substitution of the tyrosine residue for serine. **(C)** The PCR products of the *cnt1* mutant compared with its control. **(D)** Results of RT-qPCR analysis showing a relative expression of *cnt1* in control flies, *cnt1* ^*FD*^ mutant flies, and rescue flies *cnt1* ^*FD*^ *;cnt1::GFP*. Expressions were normalized to *αTub84B* and *act5C* transcripts (ΔΔCT). Significance was analyzed by one-way analysis of variance (ANOVA) and labeled as follows: ***p < 0.001, NS > 0.05; n = 4. Error bars are shown as mean ± SEM.

Using CRISPR-Cas9 technology (Kondo and Ueda, 2013), we deleted the internal part of the *cnt1* gene using two guide RNAs (gRNA) targeting the first and third coding exons (Figure 3A). The double-strand breaks resulted in a deletion of 477 bp and the substitution of an amino acid at the break site (Figures 3B,C). The deletion did not affect the reading frame of the gene but removed three transmembrane domains out of 13. We confirmed the *cnt1* mutation by sequencing and RT-qPCR (Figure 3D).

**FIGURE 4:**
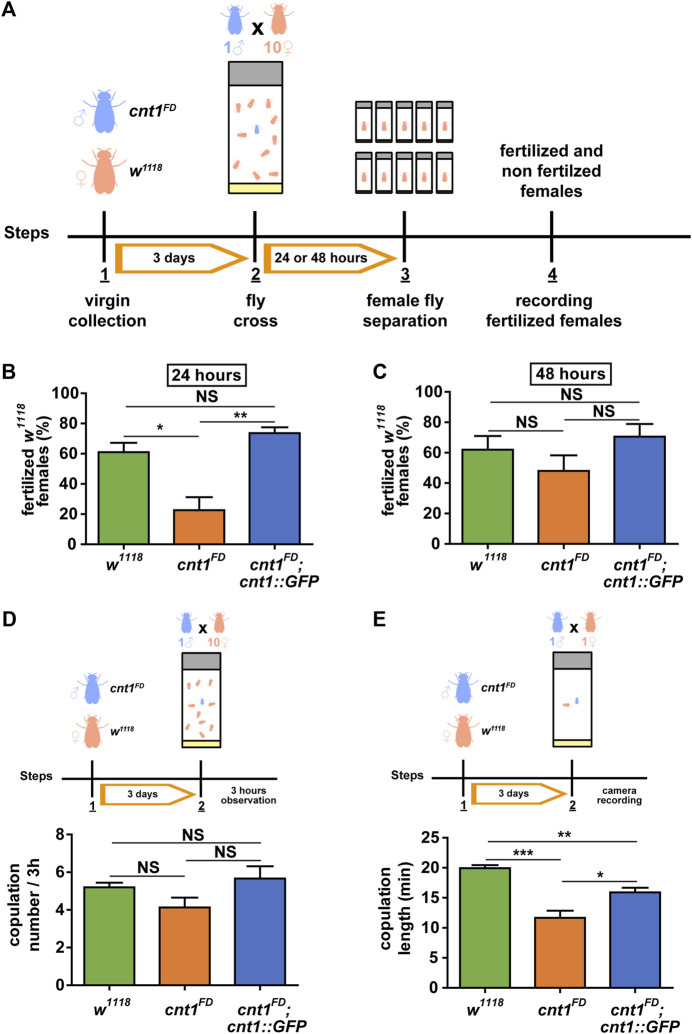
The *cnt1* mutation causes partial sterility in *Drosophila* males. **(A)** Experimental procedure of the fertility assay. Virgin mutant males (*cnt1* ^*FD*^) and control females (*w* ^*1118*^) were collected. After 3 days, one male was placed in a vial with 10 females for 24 or 48 h. Subsequently, each female was placed separately in a single vial, and the presence or absence of larvae was recorded. **(B)** Percentage of fertilized females after 24 h of mating with *cnt1* ^*FD*^ males; n ≥ 10. **(C)** Percentage of fertilized females after 48 h of mating with *cnt1* ^*FD*^ males; n ≥ 10. **(D)** Number of copulations within 3 h of observation. Virgin males (*cnt1* ^*FD*^) and females (*w* ^*1118*^) were collected. After 3 days, one male was placed in a vial with 10 females, and their mating was observed; n = 15. Error bars are presented as mean ± SEM. **(E)** Duration of copulation and experimental procedure. Virgin males (*cnt1* ^*FD*^) and females (*w* ^*1118*^) were collected. After 3 days, a male was placed in a vial with a female, and mating was recorded with a camera. The calculated times of copulations are in minutes; n = 15. Significance was analyzed by one-way ANOVA **(C)** and Kruskal–Wallis **(B,D,E)**; and labeled as follows: *p < 0.05, **p < 0.01, ***p < 0.001, NS > 0.05. Error bars are presented as mean ± SEM.

The male and female mutants showed normal viability. Given that c*nt1* expression is high in *Drosophila* testes, we investigated whether *cnt1* mutation affects male fertility. Individual naive males of *w* ^*1118*^ and *cnt1* ^*FD*^ were confined with 10 virgin *w* ^*1118*^ females for 24 or 48 h (Figure 4A). The *cnt1* ^*FD*^ males were able to fertilize three times fewer females within 24 h than the control male flies (Figure 4B). Notably, extending the mating to 48 h showed no significant difference in fertility between the *cnt1* ^*FD*^ and *w* ^*1118*^ males (Figure 4C), suggesting that *cnt1* ^*FD*^ males are less fertile and may require more time to inseminate all females.

Previous experiments have shown that 75% of the proteins tagged with GFP are functional *in vivo* (Nagarkar-Jaiswal et al., 2015). To determine whether the tagged *cnt1* was functional and test whether the observed *cnt1* ^*FD*^ phenotype was specific to *cnt1* ^*FD*^ mutation, we crossed the *cnt1* ^*FD*^ flies with flies carrying the *cnt1::GFP* transgene. As shown in Figures 4B,C, males of the rescue line *cnt1* ^*FD*^ *;cnt1::GFP* were as fertile as the control line. We also used hetero-allelic flies carrying a genomic deletion including *cnt1* and the results show that after 24 h of mating the hetero-allelic male *cnt1* ^*FD*^ */Df*(*2R*)*BSC271* flies were able to fertilize a similar percentage of females as the homozygous male *cnt1* ^*FD*^ mutants but not as many as the control male *w* ^*1118*^ flies (Supplementary Figure S3). These data confirm that the observed phenotypes are caused by the loss of *cnt1* function.

### *cnt1* affects mating behavior

Based on the above results, we tested whether the fertility disorder was related to abnormal mating behavior. To test whether mating frequency is affected by the *cnt1* mutation, we performed a test in which a male was placed in a vial with 10 virgin females and mating was recorded for 3 h. The observations showed no significant difference in mating frequency among the *cnt1* ^*FD*^ flies, *w* ^*1118*^ control flies, and *cnt1* ^*FD*^ *;cnt1::GFP* rescue flies (Figure 4D).

In the next experiment, single 3-day-old naïve males were paired with single virgin females, and their copulation time was recorded. Again, we observed that all *cnt1* ^*FD*^ males mated with the females at the same frequency, but the copulation time was shorter compared with the controls (Figure 4E). Thus, we conclude that the changes in copulation duration in *cnt1* mutants likely contribute to the observed phenotype of lower fecundity.

### *cnt1* mutation affects spermatogenesis

**FIGURE 5:**
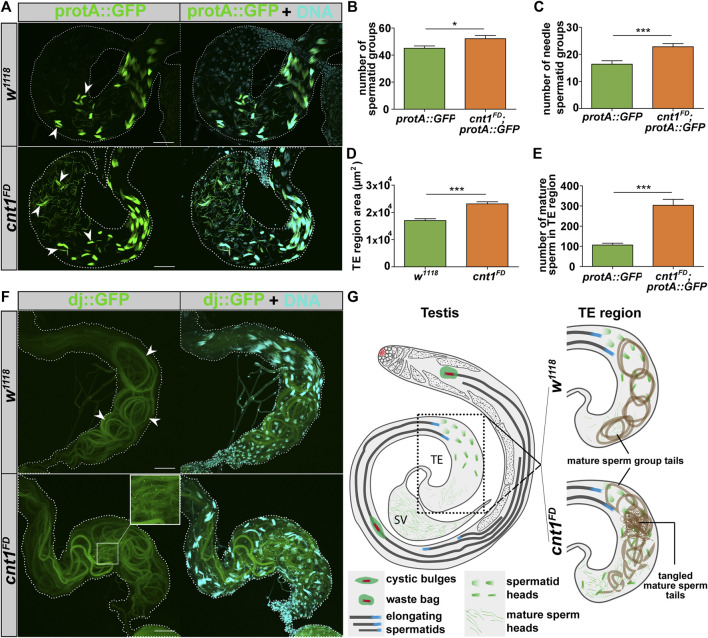
The *cnt1* mutation increases the number of spermatid groups and spermatozoa in the TE region and causes tail miscoiling. **(A)** Microscopic observation of spermatids expressing protamine A signals in the region of the terminal epithelium of the testis. The arrowheads show examples of the needle spermatid groups. *W* ^*1118*^ is used as a control. In *w* ^*1118*^, spermatid groups expressing protamine A are observed in the first half of the TE region, whereas the spermatid groups are scattered throughout the TE region in the *cnt1* ^*FD*^ mutants. Protamine A (green), DNA (cyan). Scale bar: 40 µm. **(B)** Number of spermatid groups; n ≥ 10. **(C)** Number of needle-stage spermatid groups; n ≥ 10. **(D)** Size of the TE region; n ≥ 28. **(E)** Number of mature sperm in the TE region; n ≥ 10. Significance was analyzed with a one-tailed Student’s t-test and labeled as follows: *p < 0.05, ***p < 0.001. Error bars are shown as mean ± SEM. **(F)** Microscopic observation of coiled tails of mature sperm groups expressing a “dj” signal in the region of the terminal epithelium of the testis. *W* ^*1118*^ is used as a control. In *w* ^*1118*^, the tails of the mature sperm group were well organized and coiled (white arrowhead). In *cnt1* ^*FD*^, the tails of mature sperm were poorly organized. Upon closer inspection, the tails were tangled in the region of the TE. Dj (green), DNA (cyan). Scale bar: 40 µm. **(G)** Summarized representation of protamine A and dj organization in mature sperm in *w* ^*1118*^ and *cnt1* ^*FD*^.

The maximum *cnt1* expression is detected in canoe- and needle-stage spermatids. We tested whether their numbers are affected by the *cnt1* mutation. To test whether the reduced fertility of mutant males was caused by a defect in spermatid production, we used the *protamine A* (*protA*) fluorescent marker (*protA::GFP*) (Jayaramaiah Raja and Renkawitz-Pohl, 2005) to determine spermatid and sperm counts. *ProtA* is known to be expressed in the sperm heads from the end of spermiogenesis. We also used another marker, *don Juan* (*dj*) tagged with *GFP* (*dj::GFP*) (Santel et al., 1997; Santel et al., 1998), which is present in spermatids and sperm tails, to monitor possible defects in sperm flagella. Both *protA::GFP* and *dj::GFP* were used in the background of *cnt1* ^*FD*^ mutants, as well as in its control *w* ^*1118*^. Analysis showed that, in contrast to the control, *cnt1* ^*FD*^ had a higher number of spermatid groups, including the needle-stage spermatid groups, which were scattered throughout the TE region (Figures 5A–C). The volume in this region was also increased (Figure 5D). Moreover, we counted the number of mature sperms released in the TE region and found it to be higher in *cnt1* ^*FD*^ mutants compared with the control (Figure 5E). The higher number of spermatid groups and mature sperms in the TE region of *cnt1* ^*FD*^ mutants could be due to their higher production or slow mature sperm displacement toward the SV.

To distinguish whether the increased number of mature sperms is the result of their higher production or accumulation, we investigated whether the defect of the spermatid group number occurs in newly emerged males. The microscopic analysis of the TE regions from 24-hour-old males shows that *cnt1* ^*FD*^ already produces a high number of spermatid groups (Supplementary Figure S4). Thus, the effect of *cnt1* on spermatid groups starts at an early age in adult males, possibly due to their higher production.

The alteration in spermatid group production was followed by the disorganization of the tails of the mature sperm groups in the TE region, which sometimes showed tangled tails. This phenomenon, most likely occurred during coiling (Figure 5F). In wild-type flies, the coiling process of mature sperm groups occurs with a well-defined circular pattern of flagella, whereas mature sperm groups from *cnt1* ^*FD*^ flies do not show a similar circular shape (Figures 5F,G). We also show that *cnt1* ^*FD*^ flies have a high proportion of miscoiled tails (87.8%) compared with *w* ^*1118*^ (11.76%) and *cnt1* ^*FD*^ *;cnt1::GFP* (19.44%) (Supplementary Figure S5). This could be due to insufficient space in the TE region for new ICs to proceed with the remaining steps of spermatogenesis or because the tail of the *Drosophila* mature sperm has a defect in coiling due to the *cnt1* mutation.

We also counted the number of spermatozoa in the SV to verify whether the observed tail defect in the TE region affected sperm migration. The results showed that sperm count was lower in the *cnt1* ^*FD*^ SV compared with the control. This may indicate that the fertility defect in *cnt1* ^*FD*^ males is partly due to insufficient sperm count in the SV (Supplementary Figure S6).

### *cnt1* mutation affects the apoptotic machinery during spermatid individualization

**FIGURE 6:**
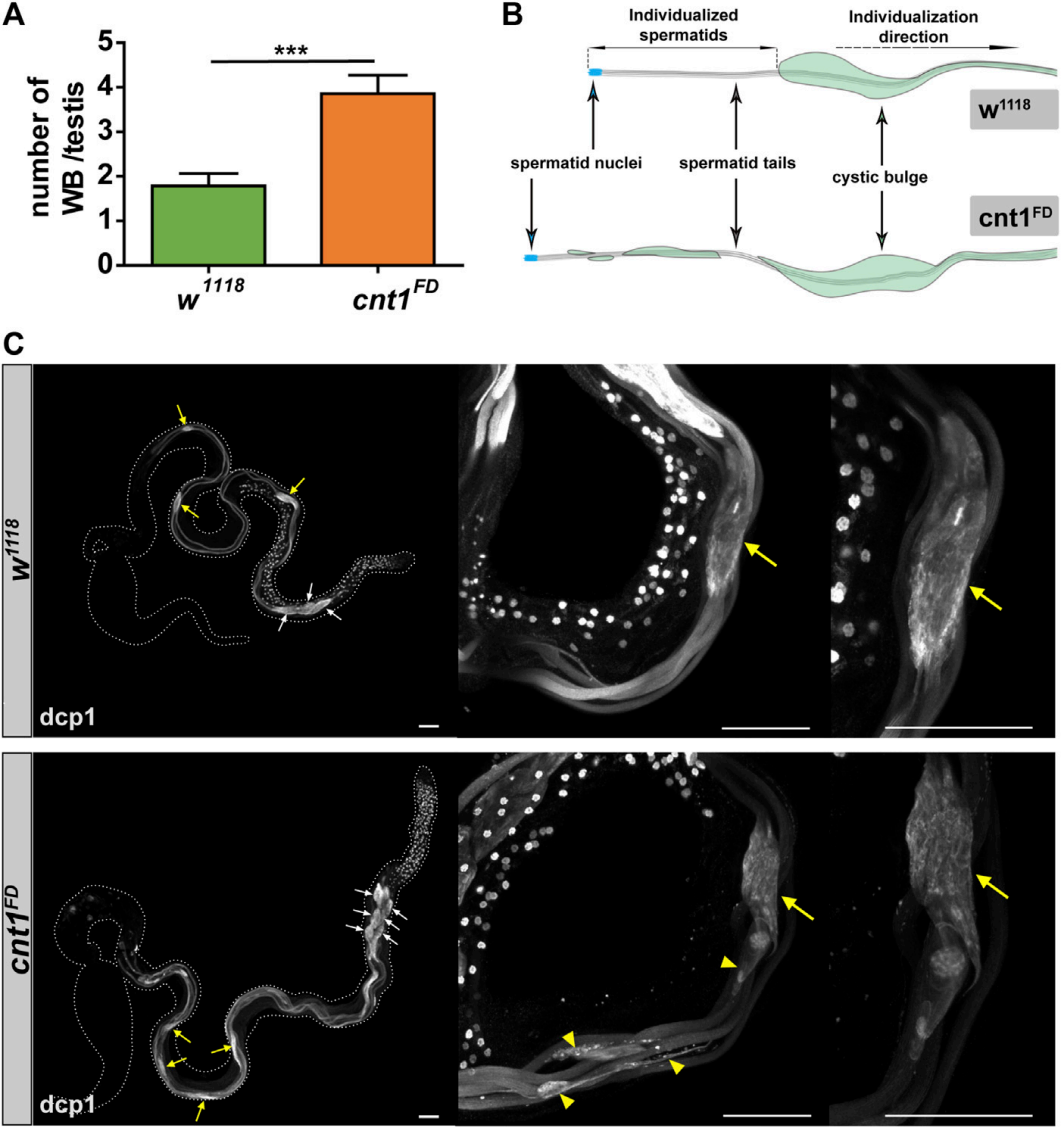
*Cnt1* mutation impairs spermatid maturation during the spermatid individualization process. **(A)** Number of waste bags in *w* ^*1118*^ and *cnt1* ^*FD*^ strains. Significance was analyzed using one-tailed Student’s t-test and labeled as follows: ***p < 0.001; n ≥ 10. Error bars are shown as mean ± SEM. **(B)** Illustration of the distorted shape of the cystic bulge in *cnt1* ^*FD*^ compared with *w* ^*1118*^. **(C)** Testis staining with death caspase-1 antibody (white). Dcp1 signal is absent in the individualized part of the cyst in *w* ^*1118*^, whereas it is present in *cnt1* mutants (yellow arrowhead). The yellow arrows point to the cystic bulges, while the white arrows point to the waste bags. Scale bar: 50 µm.

To further examine the reason for the accumulation of spermatids and the disorganization of mature sperms during coiling in the TE region of the *cnt1* mutant, we tested whether the individualization process was affected. We used the *death caspase-1 (dcp1)* antibody, which stains CB and WB. In *Drosophila*, CB and WB are formed during the individualization of elongated spermatids. They are important for the removal of redundant cytoplasm and organelles that are degraded in the bag once they reach the end of the spermatid tail (Fabian and Brill, 2012). As shown in Figure 6A, *cnt1* ^*FD*^ has more WB than the control group. In addition, most of the CB did not have an oval shape but were irregular and left a trail as they moved toward the tail end of the spermatid (Figures 6B,C). This indicates that caspase signaling remained active in the individualized portion of the spermatids (Huh et al., 2004), and that the apoptotic-like process required for the elimination of excess organelles is impaired in *cnt1* ^*FD*^ mutants. This may be related to the disorganization of the tail of spermatids observed in *cnt1* ^*FD*^ in the TE region.

### *cnt1* is required to maintain mitochondrial morphology

**FIGURE 7:**
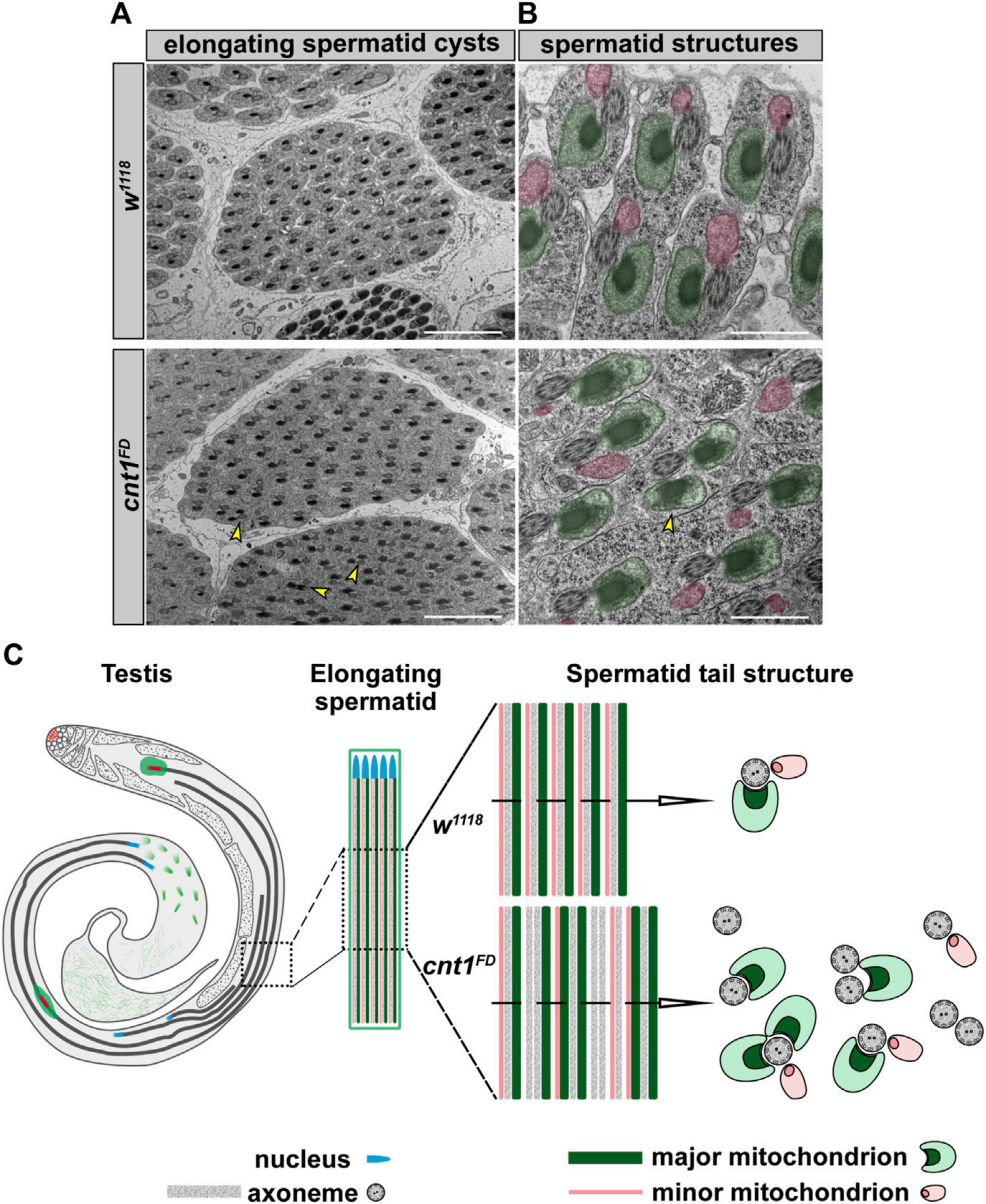
The *cnt1* mutants exhibit mitochondrial defects. **(A)** Transversal section of *w* ^*1118*^ and *cnt1* ^*FD*^ mutant testes showing a cyst of elongating spermatids. Scale bar: 5 µm. **(B)** *W* ^*1118*^ shows a normal structure of an elongated spermatid tail with one axoneme, two major mitochondria, and one minor mitochondrion. *cnt1* ^*FD*^ shows an abnormal structure of an elongated spermatid tail with one or two axonemes and an abnormal number of mitochondrial subunits. Major mitochondria (highlighted in green), minor mitochondria (highlighted in pink). Scale bar: 1 µm. **(C)** Summary of observed mitochondrial phenotypes in the *cnt1* ^*FD*^ mutant compared with *w* ^*1118*^.

To further explore the cause of the increased number of spermatids and the disorganization of their tails in the TE region, we checked the number of spermatids within their cyst group and the elongated spermatid shape under a transmission electron microscope in both control and mutant testes. This allowed us to examine the major spermatid structures, including the major and minor mitochondria and the axoneme. These components serve as structural support for the sustained elongation of the sperm tail and sperm motility in *Drosophila* (Porter, 1996; Noguchi et al., 2011). In the cross-section of the *w* ^*1118*^ testis, the spermatid cysts revealed a group of 64 spermatids. Moreover, the structure of the elongated spermatids in the *w* ^*1118*^ had all the components required to generate a healthy sperm (Figures 7A,B). By contrast, *cnt1* ^*FD*^ had a lower number of spermatids within a cyst, ranging from 47 to 64, compared to the controls (Figure 7A and Supplementary Figures S7A,B). Some of these cysts contained unusual shapes of elongated spermatids (Figures 7A,B and Supplementary Figure S7).

The male *cnt1* ^*FD*^ mutant, in contrast to *w* ^*1118*^ and *cnt1* ^*FD*^ *;cnt1::GFP*, exhibited spermatids with abnormal numbers of major and minor mitochondria (Figures 7B,C and Supplementary Figures S7A,C). In several cysts, some of the spermatid structures were in a common cytoplasm, indicating that they could not be separated from each other (Supplementary Figure S7A) (Huh et al., 2004). Fused spermatids and spermatids with altered number of mitochondria in *cnt1* ^*FD*^ were calculated and compared with those of control flies *w* ^*1118*^ and *cnt1* ^*FD*^ *;cnt1::GFP.* We show that *cnt1* ^*FD*^ mutants have an average of two defective spermatids per cyst compared with the controls (Supplementary Figure S7D). These features may account for the failed coiling process and the presence of tangled sperm tails in the TE region, where they could not move toward SV. This could be due to abnormal mitochondrial activity and other physiological changes that could affect the energy balance of the spermatids.

### RNAseq analysis and data evaluation

To investigate the gene expression profile associated with the phenotype of the *cnt1* mutant, we performed RNAseq of reproductive oragans cDNA libraries of *cnt1* ^*FD*^ mutants and the control *w* ^*1118*^ group (three biological replicates for each *cnt1* ^*FD*^ and *w* ^*1118*^ strain). RNAseq analysis identified 4,755 differentially expressed genes in the testes of the *cnt1* ^*FD*^ mutants, among which 2,472 were upregulated and 2,283 were downregulated (Supplementary Table S3). RNAseq data were validated through RT-qPCR (Supplementary Figure S8 and Supplementary Table S1). Ten representative genes were selected, and RT-qPCR showed concordant results with the RNA-sequencing data.

**FIGURE 8:**
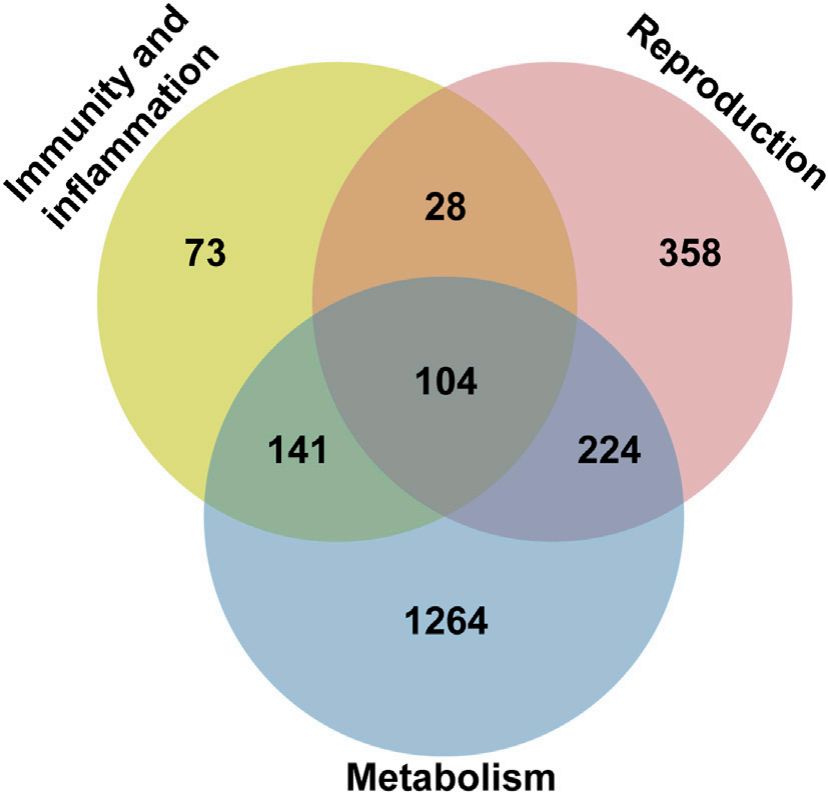
Functional classification of differentially regulated genes from the RNAseq data of *cnt1* ^*FD*^ and *w* ^*1118*^ in a Venn diagram. The pie chart shows the distribution of genes in different categories with special emphasis on the reproduction domain. The Venn diagram shows genes with adj. *p*-value ≥ 0.05.

Pathview analysis of the differentially expressed genes (adj. *p*-value<0.05) revealed only one significant KEGG pathway, namely, the ribosomal pathway (dme03010). Two protein-coding ribosomal genes were upregulated, while 78 protein-coding ribosomal genes were downregulated (Supplementary Figure S9, Supplementary Table S3). By contrast, the expressions of genes encoding ribosomal RNAs of both subunits were stable (Supplementary Figure S9). The dysregulated genes were associated with both large and small ribosomal subunits involved in cytoplasmic translation (Supplementary Figures S9, S10). Subsequently, RNA sequencing data were further filtered for abs (log2FC) > 1 and annotated with GO terms from *Drosophila melanogaster*. The genes were sorted in a Venn diagram according to three main categories: reproduction (714 genes), immunity and Inflammation (346 genes), and metabolism (1733 genes) (Figure 8 and Supplementary Table S3). Among the genes in the common category, *death regulator Nedd2-like caspase* (*dronc*), and *death-associated inhibitor of apoptosis 1* (*diap1*) were dysregulated. These genes have previously been linked to impaired apoptotic machinery during spermatid individualization (Huh et al., 2004). Data analysis also revealed altered expressions of a number of genes known from previous studies to be involved in male fertility, including *sperm-specific dynein intermediate chain 4* (*sdic4*), a dynein gene essential for sperm tail motorization (Yeh et al., 2012).

## Discussion

We have demonstrated that the *cnt1* gene, which is almost entirely expressed in the testis, is required for male fertility in *Drosophila*. We created a mutation in *cnt1* through targeted mutagenesis. The mutant showed alterations in reproductive behavior and defects in sperm maturation. Consequently, our characterization of the transcriptional responses to the *cnt1* mutation revealed changes in the expression of key genes that were previously reported to play a role in the above-mentioned aspects of male fertility.

### Mating behavior

Our data indicate that male fertility caused by the *cnt1* mutation is partly due to a behavioral defect involving a short copulation duration. Previous reports have shown that the duration of copulation in *Drosophila* is primarily controlled by males (MacBean and Parsons, 1967; Lee and Hall, 2001; Acebes et al., 2004), with a considerable period to ensure successful sperm transfer beginning 5 minutes after copulation (Gilchrist and Partridge, 2000; Crickmore and Vosshall, 2013). Abnormal copulation duration has been found in a number of mutants, including null mutants of the clock genes *period* and *timeless* (Beaver and Giebultowicz, 2004), and *fru* mutants (Lee et al., 2001). For example, *fru* mutation affect copulation by altering fru neurons. These neurons localized in the abdominal ganglion exhibit innervation at the SV, accessory gland, and end base of the testis. Interestingly, knocking down a subset of these neurons, called sAbg-1 using the *UAS-*tetanus toxin (TNT) resulted in shortened copulation duration (Jois et al., 2018).

We have previously shown that mutations in the *equilibrative nucleoside transporter 2* (*ent2*) or the *adenosine receptor* (*AdoR*) of *Drosophila* impair synaptic transmission and memory (Knight et al., 2010). Therefore, we hypothesize that the *cnt1* ^*FD*^ induced loss of adenosine nucleoside transport in the testis may also impair adenosine signaling, thus affecting behavior (Leung et al., 2001). Accordingly, a recent study has shown that phosphodiesterase pde1, an enzyme that degrades cyclic AMP (a downstream signal of the adenosine receptor), causes male sterility and behavioral abnormalities that affect copulation in *Drosophila* (Morton et al., 2010). The adenosine pathway is known to cause neuromodulation in mammals and has been suggested to influence sex-dependent neuropsychiatric behavior and erection in humans (Phatarpekar et al., 2010; Osborne et al., 2018).

### Sperm maturation

Our results further showed that the effects of the *cnt1* ^*FD*^ mutation are pleiotropic and include morphological changes during spermiogenesis, where spermatid maturation is defective. The most striking phenotype of the *cnt1* mutation is a higher number of spermatid groups and mature sperms (Figure 5). We also showed that the tail coiling of mature sperm groups was disorganized and tangled, which probably prevented them from moving to the SV, where the sperm count was low. The higher number of spermatid groups contrasts with the low number of mature sperms in the SV, suggesting that spermatids accumulate in the TE region.

Thus, *cnt1* appears to play an important role in regulating mechanical movements in maturing sperms, which reportedly depend on intact mitochondria and dynein proteins (Ranz et al., 2003; Yeh et al., 2012), the latter of which require high ATP concentrations for proper function (Xie et al., 2006). A previous study has shown that knockdown of ATP synthase results in male infertility and abnormal spermatogenesis in *Drosophila* testes (Yu et al., 2019). In addition, ATP synthase has been reported to affect the elongated shape of mitochondria when knocked out (Sawyer et al., 2017). This finding is consistent with our RNAseq data that shows the dysregulation of genes belonging to the mitochondrial respiratory chain (*ATP synthase-coupling factor 6* (*ATPsyn-Cf6*), *NADH dehydrogenase 75* (*ND-75*)*, cytochrome-c1* (*cyt-c1*), and *cytochrome c distal* (*cyt-c-d*)), of which *cyt-c1* was confirmed by RT-qPCR to be downregulated in *cnt1* ^*FD*^ mutants (Supplementary Figure S8). In addition, previous studies have shown that mutations in dynein, such as *dic61B*, play a crucial role in sperm motility (Fatima, 2011). The dynein mutation in *dic61B* has been associated with impaired spermatid individualization and motility, which eventually led to male sterility. Furthermore, the *dic61B* mutation has also been reported to contribute to a defect in the formation of major and minor mitochondria derivatives (Fatima, 2011), supporting the idea that alterations in both dynein and mitochondria may also affect sperm axoneme movement in *cnt1* ^*FD*^ mutants. Among the dysregulated dynein genes in our RNAseq data, we found and confirmed that the gene-encoding sperm-specific dynein (*sdic4*) is upregulated in *cnt1* ^*FD*^ mutants. Taken together, mitochondria and dynein functions may be altered in *cnt1* ^*FD*^ mutants.

In addition, our data showed an increased number of waste bags, consistent with the observed high number of spermatid groups and low count of mature sperms in the TE region. Furthermore, these bags left a trail as they moved to the end of the spermatid tail, suggesting that the bags could not process their degradation properly. A similar phenotype to the *cnt1* ^*FD*^ mutant was previously observed in the *driceless* mutant and associated with unusual active caspase signaling (Huh et al., 2004). Further phenotypic analysis using electron microscopy confirmed that, similar to *driceless* mutants (Huh et al., 2004), the spermatid cysts in *cnt1* ^*FD*^ underwent partial individualization, with the spermatid cysts containing sheathed spermatids in a common cytoplasmic membrane, while the other spermatids have their own cytoplasmic membrane. These mutants also showed a defect in the cystic bulges with trailing edge (Huh et al., 2004). Consistently, our RNAseq data show a dysregulation of a key gene involved in the apoptotic machinery during spermatid individualization: *dcp1* (Huh et al., 2004). It was reported in connection with the failure of the individualization process, like the *cnt1* ^*FD*^ mutant, in which multiple cysts contained many single spermatid units with excess cytoplasm.

*Drosophila cnt1* and *cnt2* are similar genes which most probably have the same biochemical function (nucleoside transport) but they differ by tissue specificity of expression. Their phenotypes seem to be quite independent. In our experiments, the removal of one copy of *cnt2* did not enhance the *cnt1* fertility phenotype the heteroallelic flies *cnt1* ^*FD*^ */Df*(*2R*)*BSC271* (this deletion lacks both *cnt1* and *cnt2*) (Supplementary Figure S3).

Similar to *Drosophila*, the expressions of Cnt isoforms were detected in both human and rat testes, mainly in the Sertoli cells, which are essential for sperm nutrition (Klein et al., 2013; Hau et al., 2020). The pharmacological blockade of nucleoside transporters in Sertoli cells seems to interfere with the spermatid maturation process, suggesting that nucleoside transport may be important for providing the nucleosides essential for sperm maturation (Leung et al., 2001; Kato et al., 2005). Previous research in mice has shown the relationship between sperm motility and mitochondrial dysfunction (Cardullo and Baltz, 1991; Ruiz-Pesini et al., 1998). These findings argue for similarities in the regulation of spermatogenesis in *Drosophila* and mammals.

In summary, we show that *cnt1* has a pleiotropic effect on mating behavior, spermatid maturation, and spermatid mitochondrial morphology in *Drosophila*. It affects the transcription of several key genes known from previous reports to be involved in these phenotypes, including dynein, ATP synthases, and apoptotic genes. Further work is needed to understand the relationship of these *cnt1* phenotypes to adenosine signaling and related metabolic pathways.

## Data Availability

The datasets presented in this study can be found in online repositories. The names of the repository/repositories and accession number(s) can be found below: SRA data: PRJNA838856, https://www.ncbi.nlm.nih.gov/sra/PRJNA838856.

## References

[B1] Acebes A. Grosjean Y. Everaerts C. Ferveur J. F. (2004). Cholinergic control of synchronized seminal emissions in *Drosophila* . Curr. Biol. 14, 704–710. 10.1016/j.cub.2004.04.003 15084286

[B2] Alzyoud E. Vedelek V. Réthi-Nagy Z. Lipinszki Z. Sinka R. (2021). Microtubule organizing centers contain testis-specific γ-turc proteins in spermatids of *Drosophila* . Front. Cell Dev. Biol. 9, 727264. 10.3389/fcell.2021.727264 34660584 PMC8511327

[B3] Augière C. Lapart J.-A. Duteyrat J.-L. Cortier E. Maire C. Thomas J. (2019). salto/CG13164 is required for sperm head morphogenesis in *Drosophila* . Mol. Biol. Cell 30, 636–645. 10.1091/mbc.E18-07-0429 30601696 PMC6589691

[B4] Bader M. Arama E. Steller H. (2010). A novel F-box protein is required for caspase activation during cellular remodeling in *Drosophila* . Development 137, 1679–1688. 10.1242/dev.050088 20392747 PMC2860250

[B5] Baker B. S. Taylor B. J. Hall J. C. (2001). Are complex behaviors specified by dedicated regulatory genes? Reasoning from *Drosophila* . Cell 105, 13–24. 10.1016/S0092-8674(01)00293-8 11300999

[B6] Baptissart M. Vega A. Martinot E. Volle D. H. (2013). Male fertility: Is spermiogenesis the critical step for answering biomedical issues? Spermatogenesis 3, e24114. 10.4161/spmg.24114 23885302 PMC3710220

[B7] Batut B. Hiltemann S. Bagnacani A. Baker D. Bhardwaj V. Blank C. (2018). Community-driven data analysis training for biology. Cell Syst. 6, 752–758.e1. 10.1016/j.cels.2018.05.012 29953864 PMC6296361

[B8] Beaver L. M. Giebultowicz J. M. (2004). Regulation of copulation duration by period and timeless in *Drosophila melanogaster* . Curr. Biol. 14, 1492–1497. 10.1016/j.cub.2004.08.022 15324667

[B9] Cardullo R. A. Baltz J. M. (1991). Metabolic regulation in mammalian sperm: Mitochondrial volume determines sperm length and flagellar beat frequency. Cell Motil. Cytoskelet. 19, 180–188. 10.1002/cm.970190306 1878988

[B10] Chen X. Hiller M. Sancak Y. Fuller M. T. (2005). Tissue-specific TAFs counteract polycomb to turn on terminal differentiation. Science 310, 869–872. 10.1126/science.1118101 16272126

[B11] Chintapalli V. R. Wang J. Dow J. A. T. (2007). Using FlyAtlas to identify better *Drosophila melanogaster* models of human disease. Nat. Genet. 39, 715–720. 10.1038/ng2049 17534367

[B12] Clark K. Karsch-Mizrachi I. Lipman D. J. Ostell J. Sayers E. W. (2016). GenBank. Nucleic Acids Res. 44, D67–D72. 10.1093/nar/gkv1276 26590407 PMC4702903

[B13] Crickmore M. A. Vosshall L. B. (2013). Opposing dopaminergic and GABAergic neurons control the duration and persistence of copulation in *Drosophila* . Cell 155, 881–893. 10.1016/j.cell.2013.09.055 24209625 PMC4048588

[B14] Demarco R. S. Eikenes Å. H. Haglund K. Jones D. L. (2014). Investigating spermatogenesis in *Drosophila melanogaster* . Methods 68, 218–227. 10.1016/j.ymeth.2014.04.020 24798812 PMC4128239

[B15] Dos Santos-Rodrigues A. Pereira M. R. Brito R. de Oliveira N. A. Paes-de-Carvalho R. (2015). Adenosine transporters and receptors: Key elements for retinal function and neuroprotection. Vitam. Horm. 98, 487–523. 10.1016/bs.vh.2014.12.014 25817878

[B16] Edgar R. C. (2004). MUSCLE: Multiple sequence alignment with high accuracy and high throughput. Nucleic Acids Res. 32, 1792–1797. 10.1093/nar/gkh340 15034147 PMC390337

[B17] Ejsmont R. K. Ahlfeld P. Pozniakovsky A. Stewart A. F. Tomancak P. Sarov M. (2011). Recombination-mediated genetic engineering of large genomic DNA transgenes. Methods Mol. Biol. 772, 445–458. 10.1007/978-1-61779-228-1_26 22065454

[B18] Ejsmont R. K. Sarov M. Winkler S. Lipinski K. A. Tomancak P. (2009). A toolkit for high-throughput, cross-species gene engineering in *Drosophila* . Nat. Methods 6, 435–437. 10.1038/nmeth.1334 19465918

[B19] Fabian L. Brill J. A. (2012). *Drosophila* spermiogenesis: Big things come from little packages. Spermatogenesis 2, 197–212. 10.4161/spmg.21798 23087837 PMC3469442

[B20] Fatima R. (2011). *Drosophila* Dynein intermediate chain gene, Dic61B, is required for spermatogenesis. PLoS One 6, e27822. 10.1371/journal.pone.0027822 22145020 PMC3228723

[B21] Fleischmannova J. Kucerova L. Sandova K. Steinbauerova V. Broz V. Simek P. (2012). Differential response of *Drosophila* cell lines to extracellular adenosine. Insect biochem. Mol. Biol. 42, 321–331. 10.1016/j.ibmb.2012.01.002 22266077

[B22] Gilchrist A. S. Partridge L. (2000). Why it is difficult to model sperm displacement in *Drosophila melanogaster*: The relation between sperm transfer and copulation duration. Evolution 54, 534–542. 10.1111/j.0014-3820.2000.tb00056.x 10937230

[B23] Gray J. H. Owen R. P. Giacomini K. M. (2004). The concentrative nucleoside transporter family, SLC28. Pflugers Arch. 447, 728–734. 10.1007/s00424-003-1107-y 12856181

[B24] Groth A. C. Fish M. Nusse R. Calos M. P. (2004). Construction of transgenic *Drosophila* by using the site-specific integrase from phage phiC31. Genetics 166, 1775–1782. 10.1534/genetics.166.4.1775 15126397 PMC1470814

[B25] Guindon S. Dufayard J.-F. Lefort V. Anisimova M. Hordijk W. Gascuel O. (2010). New algorithms and methods to estimate maximum-likelihood phylogenies: Assessing the performance of PhyML 3.0. Syst. Biol. 59, 307–321. 10.1093/sysbio/syq010 20525638

[B26] Hau R. K. Miller S. R. Wright S. H. Cherrington N. J. (2020). Generation of a hTERT-immortalized human Sertoli cell model to study transporter dynamics at the blood-testis barrier. Pharmaceutics 12, E1005. 10.3390/pharmaceutics12111005 33105674 PMC7690448

[B27] Huh J. R. Vernooy S. Y. Yu H. Yan N. Shi Y. Guo M. (2004). Multiple apoptotic caspase cascades are required in nonapoptotic roles for *Drosophila* spermatid individualization. PLoS Biol. 2, E15. 10.1371/journal.pbio.0020015 14737191 PMC300883

[B28] Jayaramaiah Raja S. Renkawitz-Pohl R. (2005). Replacement by *Drosophila melanogaster* protamines and Mst77F of histones during chromatin condensation in late spermatids and role of sesame in the removal of these proteins from the male pronucleus. Mol. Cell. Biol. 25, 6165–6177. 10.1128/MCB.25.14.6165-6177.2005 15988027 PMC1168805

[B29] Jois S. Chan Y. B. Fernandez M. P. Leung A. K.-W. (2018). Characterization of the sexually dimorphic fruitless neurons that regulate copulation duration. Front. Physiol. 9, 780. 10.3389/fphys.2018.00780 29988589 PMC6026680

[B30] Kato R. Maeda T. Akaike T. Tamai I. (2005). Nucleoside transport at the blood-testis barrier studied with primary-cultured Sertoli cells. J. Pharmacol. Exp. Ther. 312, 601–608. 10.1124/jpet.104.073387 15547112

[B31] Klein D. M. Evans K. K. Hardwick R. N. Dantzler W. H. Wright S. H. Cherrington N. J. (2013). Basolateral uptake of nucleosides by Sertoli cells is mediated primarily by equilibrative nucleoside transporter 1. J. Pharmacol. Exp. Ther. 346, 121–129. 10.1124/jpet.113.203265 23639800 PMC3684844

[B32] Klein D. M. Harding M. C. Crowther M. K. Cherrington N. J. (2017). Localization of nucleoside transporters in rat epididymis. J. Biochem. Mol. Toxicol. 31, e21911. 10.1002/jbt.21911 28322028

[B33] Knight D. Harvey P. J. Iliadi K. G. Klose M. K. Iliadi N. Dolezelova E. (2010). Equilibrative nucleoside transporter 2 regulates associative learning and synaptic function in *Drosophila* . J. Neurosci. 30, 5047–5057. 10.1523/JNEUROSCI.6241-09.2010 20371825 PMC6632785

[B34] Kondo S. Ueda R. (2013). Highly improved gene targeting by germline-specific Cas9 expression in *Drosophila* . Genetics 195, 715–721. 10.1534/genetics.113.156737 24002648 PMC3813859

[B35] Kumar S. Stecher G. Li M. Knyaz C. Tamura K. (2018). MEGA X: Molecular evolutionary genetics analysis across computing platforms. Mol. Biol. Evol. 35, 1547–1549. 10.1093/molbev/msy096 29722887 PMC5967553

[B36] Larkin A. Marygold S. J. Antonazzo G. Attrill H. dos Santos G. Garapati P. V. (2021). FlyBase: Updates to the *Drosophila melanogaster* knowledge base. Nucleic Acids Res. 49, D899–D907. 10.1093/nar/gkaa1026 33219682 PMC7779046

[B37] Leatherman J. L. Dinardo S. (2008). Zfh-1 controls somatic stem cell self-renewal in the *Drosophila* testis and nonautonomously influences germline stem cell self-renewal. Cell Stem Cell 3, 44–54. 10.1016/j.stem.2008.05.001 18593558 PMC2601693

[B38] Lee G. Hall J. C. (2001). Abnormalities of male-specific FRU protein and serotonin expression in the CNS of fruitless mutants in *Drosophila* . J. Neurosci. 21, 513–526. 10.1523/JNEUROSCI.21-02-00513.2001 11160431 PMC6763814

[B39] Lee G. Villella A. Taylor B. J. Hall J. C. (2001). New reproductive anomalies in fruitless-mutant *Drosophila* males: Extreme lengthening of mating durations and infertility correlated with defective serotonergic innervation of reproductive organs. J. Neurobiol. 47, 121–149. 10.1002/neu.1021 11291102

[B40] Lefort V. Longueville J.-E. Gascuel O. (2017). SMS: Smart model selection in PhyML. Mol. Biol. Evol. 34, 2422–2424. 10.1093/molbev/msx149 28472384 PMC5850602

[B41] Leung G. P. Ward J. L. Wong P. Y. Tse C. M. (2001). Characterization of nucleoside transport systems in cultured rat epididymal epithelium. Am. J. Physiol. Cell Physiol. 280, C1076–C1082. 10.1152/ajpcell.2001.280.5.C1076 11287319

[B42] Lin Y.-H. Maaroufi H. O. Ibrahim E. Kucerova L. Zurovec M. (2019). Expression of human mutant huntingtin protein in *Drosophila* hemocytes impairs immune responses. Front. Immunol. 10, 2405. 10.3389/fimmu.2019.02405 31681295 PMC6805700

[B43] Lin Y.-H. Maaroufi H. O. Kucerova L. Rouhova L. Filip T. Zurovec M. (2021). Adenosine receptor and its downstream targets, mod(mdg4) and Hsp70, work as a signaling pathway modulating cytotoxic damage in *Drosophila* . Front. Cell Dev. Biol. 9, 651367. 10.3389/fcell.2021.651367 33777958 PMC7994771

[B44] Lindsley D. I. Tokuyasu K. T. (1980). “Spermatogenesis,” in Genetics and Biology of Drosophila (New York: Academic Press), 225–294.

[B45] Lotti F. Maggi M. (2018). Sexual dysfunction and male infertility. Nat. Rev. Urol. 15, 287–307. 10.1038/nrurol.2018.20 29532805

[B46] MacBean I. T. Parsons P. A. (1967). Directional selection for duration of copulation in *Drosophila melanogaster* . Genetics 56, 233–239. 10.1093/genetics/56.2.233 6040493 PMC1211499

[B47] Machado J. Abdulla P. Hanna W. J. B. Hilliker A. J. Coe I. R. (2007). Genomic analysis of nucleoside transporters in Diptera and functional characterization of DmENT2, a *Drosophila* equilibrative nucleoside transporter. Physiol. Genomics 28, 337–347. 10.1152/physiolgenomics.00087.2006 17090699

[B48] Masino S. Boison D. (2013). Adenosine: A key link between metabolism and brain activity. New York, NY: Springer. 10.1007/978-1-4614-3903-5

[B49] Molina-Arcas M. Pastor-Anglada M. (2013). Nucleoside transporters (SLC28 and SLC29) family. Pharmacogenomics of Human Drug Transporters. 11, 243–270. 10.1002/9781118353240.ch11

[B50] Morton D. B. Clemens-Grisham R. Hazelett D. J. Vermehren-Schmaedick A. (2010). Infertility and male mating behavior deficits associated with Pde1c in *Drosophila melanogaster* . Genetics 186, 159–165. 10.1534/genetics.110.118018 20551439 PMC2940284

[B51] Nagarkar-Jaiswal S. Lee P.-T. Campbell M. E. Chen K. Anguiano-Zarate S. Cantu Gutierrez M. (2015). A library of MiMICs allows tagging of genes and reversible, spatial and temporal knockdown of proteins in *Drosophila* . Elife 4, e05338. 10.7554/eLife.05338 PMC4379497 25824290

[B52] Ng C. L. Qian Y. Schulz C. (2019). Notch and Delta are required for survival of the germline stem cell lineage in testes of *Drosophila melanogaster* . PLoS One 14, e0222471. 10.1371/journal.pone.0222471 31513679 PMC6742463

[B53] Noguchi T. Koizumi M. Hayashi S. (2011). Sustained elongation of sperm tail promoted by local remodeling of giant mitochondria in *Drosophila* . Curr. Biol. 21, 805–814. 10.1016/j.cub.2011.04.016 21549602

[B54] Noguchi T. Miller K. G. (2003). A role of actin dynamics in individualization during spermatogenesis in *Drosophila melanogaster* . Development 130, 1805–1816. 10.1242/dev.00406 12642486

[B55] Osborne D. M. Sandau U. S. Jones A. T. Vander Velden J. W. Weingarten A. M. Etesami N. (2018). Developmental role of adenosine kinase for the expression of sex-dependent neuropsychiatric behavior. Neuropharmacology 141, 89–97. 10.1016/j.neuropharm.2018.08.025 30145320 PMC6867705

[B56] Pavlou H. J. Goodwin S. F. (2013). Courtship behavior in *Drosophila melanogaster*: Towards a ‘courtship connectome. Curr. Opin. Neurobiol. 23, 76–83. 10.1016/j.conb.2012.09.002 23021897 PMC3563961

[B57] Pérez-Torras S. Mata-Ventosa A. Drögemöller B. Tarailo-Graovac M. Meijer J. Meinsma R. (2019). Deficiency of perforin and hCNT1, a novel inborn error of pyrimidine metabolism, associated with a rapidly developing lethal phenotype due to multi-organ failure. Biochim. Biophys. Acta. Mol. Basis Dis. 1865, 1182–1191. 10.1016/j.bbadis.2019.01.013 30658162

[B58] Phatarpekar P. V. Wen J. Xia Y. (2010). Role of adenosine signaling in penile erection and erectile disorders. J. Sex. Med. 7, 3553–3564. 10.1111/j.1743-6109.2009.01555.x 19889148 PMC2906687

[B59] Porter M. E. (1996). Axonemal dyneins: Assembly, organization, and regulation. Curr. Opin. Cell Biol. 8, 10–17. 10.1016/S0955-0674(96)80042-1 8791407

[B60] Ranz J. M. Ponce A. R. Hartl D. L. Nurminsky D. (2003). Origin and evolution of a new gene expressed in the *Drosophila* sperm axoneme. Genetica 118, 233–244. 10.1023/A:1024186516554 12868612

[B61] Resende L. P. F. Boyle M. Tran D. Fellner T. Jones D. L. (2013). Headcase promotes cell survival and niche maintenance in the *Drosophila* testis. PLoS One 8, e68026. 10.1371/journal.pone.0068026 23874487 PMC3706621

[B62] Rideout E. J. Billeter J.-C. Goodwin S. F. (2007). The sex-determination genes fruitless and doublesex specify a neural substrate required for courtship song. Curr. Biol. 17, 1473–1478. 10.1016/j.cub.2007.07.047 17716899 PMC2583281

[B63] Ruiz-Pesini E. Diez C. Lapeña A. C. Pérez-Martos A. Montoya J. Alvarez E. (1998). Correlation of sperm motility with mitochondrial enzymatic activities. Clin. Chem. 44, 1616–1620. 10.1093/clinchem/44.8.1616 9702947

[B64] Santel A. Blümer N. Kämpfer M. Renkawitz-Pohl R. (1998). Flagellar mitochondrial association of the male-specific Don Juan protein in *Drosophila* spermatozoa. J. Cell Sci. 111 (2), 3299–3309. 10.1242/jcs.111.22.3299 9788872

[B65] Santel A. Winhauer T. Blümer N. Renkawitz-Pohl R. (1997). The *Drosophila* don juan (dj) gene encodes a novel sperm specific protein component characterized by an unusual domain of a repetitive amino acid motif. Mech. Dev. 64, 19–30. 10.1016/s0925-4773(97)00031-2 9232593

[B66] Sarov M. Barz C. Jambor H. Hein M. Y. Schmied C. Suchold D. (2016). A genome-wide resource for the analysis of protein localisation in *Drosophila* . Elife 5, e12068. 10.7554/eLife.12068 26896675 PMC4805545

[B67] Sawyer E. M. Brunner E. C. Hwang Y. Ivey L. E. Brown O. Bannon M. (2017). Testis-specific ATP synthase peripheral stalk subunits required for tissue-specific mitochondrial morphogenesis in *Drosophila* . BMC Cell Biol. 18, 16. 10.1186/s12860-017-0132-1 28335714 PMC5364652

[B68] Schneider C. A. Rasband W. S. Eliceiri K. W. (2012). NIH image to ImageJ: 25 years of image analysis. Nat. Methods 9, 671–675. 10.1038/nmeth.2089 22930834 PMC5554542

[B69] Siegel R. W. Hall J. C. (1979). Conditioned responses in courtship behavior of normal and mutant *Drosophila* . Proc. Natl. Acad. Sci. U. S. A. 76, 3430–3434. 10.1073/pnas.76.7.3430 16592682 PMC383839

[B70] Soulavie F. Piepenbrock D. Thomas J. Vieillard J. Duteyrat J.-L. Cortier E. (2014). Hemingway is required for sperm flagella assembly and ciliary motility in *Drosophila* . Mol. Biol. Cell 25, 1276–1286. 10.1091/mbc.e13-10-0616 24554765 PMC3982993

[B71] Steinhauer J. (2015). Separating from the pack: Molecular mechanisms of *Drosophila* spermatid individualization. Spermatogenesis 5, e1041345. 10.1080/21565562.2015.1041345 26413413 PMC4581072

[B72] Tokuyasu K. T. (1975). Dynamics of spermiogenesis in *Drosophila melanogaster*. VI. Significance of “onion” nebenkern formation. J. Ultrastruct. Res. 53, 93–112. 10.1016/S0022-5320(75)80089-X 810602

[B73] Vedelek V. Bodai L. Grézal G. Kovács B. Boros I. M. Laurinyecz B. (2018). Analysis of *Drosophila melanogaster* testis transcriptome. BMC Genomics 19, 697. 10.1186/s12864-018-5085-z 30249207 PMC6154878

[B74] Wakimoto B. T. Lindsley D. L. Herrera C. (2004). Toward a comprehensive genetic analysis of male fertility in *Drosophila melanogaster* . Genetics 167, 207–216. 10.1534/genetics.167.1.207 15166148 PMC1470876

[B75] White-Cooper H. (2004). Spermatogenesis: Analysis of meiosis and morphogenesis. Methods Mol. Biol. 247, 45–75. 10.1385/1-59259-665-7:45 14707342

[B76] Witt E. Benjamin S. Svetec N. Zhao L. (2019). Testis single-cell RNA-seq reveals the dynamics of de novo gene transcription and germline mutational bias in *Drosophila* . Elife 8, e47138. 10.7554/eLife.47138 31418408 PMC6697446

[B77] Xie P. Dou S.-X. Wang P.-Y. (2006). Model for unidirectional movement of axonemal and cytoplasmic dynein molecules. Acta Biochim. Biophys. Sin. 38, 711–724. 10.1111/j.1745-7270.2006.00223.x 17033718

[B78] Yeh S.-D. Do T. Abbassi M. Ranz J. M. (2012). Functional relevance of the newly evolved sperm dynein intermediate chain multigene family in *Drosophila melanogaster* males. Commun. Integr. Biol. 5, 462–465. 10.4161/cib.21136 23181161 PMC3502208

[B79] Young J. D. (2016). The SLC28 (CNT) and SLC29 (ENT) nucleoside transporter families: A 30-year collaborative odyssey. Biochem. Soc. Trans. 44, 869–876. 10.1042/BST20160038 27284054

[B80] Young J. D. Yao S. Y. M. Baldwin J. M. Cass C. E. Baldwin S. A. (2013). The human concentrative and equilibrative nucleoside transporter families, SLC28 and SLC29. Mol. Asp. Med. 34, 529–547. 10.1016/j.mam.2012.05.007 23506887

[B81] Yu J. Chen B. Zheng B. Qiao C. Chen X. Yan Y. (2019). ATP synthase is required for male fertility and germ cell maturation in *Drosophila* testes. Mol. Med. Rep. 19, 1561–1570. 10.3892/mmr.2019.9834 30628672 PMC6390039

[B82] Yuan X. Zheng H. Su Y. Guo P. Zhang X. Zhao Q. (2019). *Drosophila* Pif1A is essential for spermatogenesis and is the homolog of human CCDC157, a gene associated with idiopathic NOA. Cell Death Dis. 10, 125. 10.1038/s41419-019-1398-3 30741974 PMC6370830

[B83] Zhang S. D. Odenwald W. F. (1995). Misexpression of the white (w) gene triggers male-male courtship in *Drosophila* . Proc. Natl. Acad. Sci. U. S. A. 92, 5525–5529. 10.1073/pnas.92.12.5525 7777542 PMC41728

[B84] Zhao J. Klyne G. Benson E. Gudmannsdottir E. White-Cooper H. Shotton D. (2010). FlyTED: The *Drosophila* testis gene expression database. Nucleic Acids Res. 38, D710–D715. 10.1093/nar/gkp1006 19934263 PMC2808924

[B85] Ziegler A. B. Berthelot-Grosjean M. Grosjean Y. (2013). The smell of love in *Drosophila* . Front. Physiol. 4, 72. 10.3389/fphys.2013.00072 23576993 PMC3617446

