# Supplementary material for: Mutation in Drosophila concentrative nucleoside transporter 1 alters spermatid maturation and mating behavior
Source: Front Cell Dev Biol. 2022 Aug 23;10:945572. doi: 10.3389/fcell.2022.945572 (PMC9467524; doi:10.3389/fcell.2022.945572)
Supplement: Supplementary file 3 [file DataSheet1.pdf]

frontiers

Supplementary Material

Species name	Accession number	gene	Protein sequence
1. D.melanogaster	_A1Z7N3	CNT1	-----------------MAEPIEGEQEEKPP---------------------------PSRAKRITYLVLHVLLHIIFISYFTAATIIF
2. D.erecta	_B3N816	CNT1	-----------------MADPQGGELEEKPP---------------------------PSRTERITYLVLHVLLHVIFISYFTAATIIF
3. D.yakuba	_B4P3W2	CNT1	-----------------MADPQVDELEENPP---------------------------PSRAKRITYLVLHVLLHVIFISYFTAATIIF
4. D.sechellia	_B4HSE4	CNT1	-----------------MADPAEGELDEKPP---------------------------PSRAQRITYVILHVLLHVVFISYFTAATIIF
5. D.simulans	_B4QGR1	CNT1	-----------------MADPAEGELDEKPP---------------------------PSRAQRIIYVILHVLLHVVFISYFTAATIIF
6. D.ananassae	_B3MI82	CNT1	-----------------MAEPAPAEESEEKP---------------------------KPRWRRILERVILIFIHLLFIAYFIAATVIY
7. D.willistoni	_B4MUJ0	CNT1	-------------------MDESANIEEKPEK--------------------------PSRKKKFLILLLHVIFHLILISYFIATSVVY
8. D.guanche	_A0A3B0JGG2	CNT1	----------------------MEPLDPEEE---------------------------PSKKRALPKLLLHIFFHILLISYFIAATVVY
9. D.persimilis	_B4GHY4	CNT1	----------------------MEPLEDEEV---------------------------PSKKRKILLLLLHILFHILWISYFIAATVVY
10. D.ficusphila	_A0A1W4UJK1	CNT1	---------------MDNLAPEQQNPEEKPP---------------------------RSKARRIVYLILHVLFHFLFILYFVAATVSY
11. D.melanogaster	_Q7K4A1	CNT2	---MSAESSKGAINNGYELDHKELDIRDSEEFTEHPLDDTSEV------ANKKGYFEKNPKVARLVRISIYVLLHLCVVGYFSYAT--Y
12. D.erecta	_B3N817	CNT2	---MSAESSKGSINNGYELDHKELDIRDSEEFKEIPLENISEV------PNKRGYFEKNPKVARLVRISIYVFLHLFVVGYFSYAT--Y
13. D.yakuba	_B4P3W1	CNT2	---MSAESSKGALNNGYELDHKELDIRDSEEYKEFPLEDLSED------PNKKGYFEKNPKVARLVKISIYVLLHLCVVGYFSYAT--Y
14. D.sechellia	_B4HSE3	CNT2	---MSAESSKGAINNGYELDHKELDIRDSEEFKELPLDDTSDA------PNKKGYFEKNPKVARLVRISLYVLLHLCVVGYFSYAT--Y
15. D.simulans	_B4QGR0	CNT2	---MSAESSKGAINNGYELDHKELDIRDSEEFKELPLDDTSDA------PNKKGYFEKNPKVARLVRISIYVLLHLCVVGYFSYAT--Y
16. D.ananassae	_B3MI83	CNT2	---MTDTSSIGAINNGYETDHKELSIPETEQFKELPPIGSTEK------APKPSYWDNNPKVKRIVKISLYVFLHLVVVGYFSYAT--Y
17. D.willistoni	_B4N641	CNT2	-------MEKGAINNGFELDQKDLTNKNDDLYETDVPVEILTT------QNGDLAQDNKAKITKWIKTFLKYFIHIVVIGYFSYAA--Y
18. D.guanche	_A0A3B0JP57	CNT2	---MTTDTSKGAINNGYELDHSELDIASVEPFKEIPQDGL-ELNLPRKMEDEKGFFNQNPKIARIVRISLYILLHLVVVGYFSYAT--Y
19. D.persimilis	_B4GHY3	CNT2	---MTTDTAKDVINNGYELDHRELEIPNSEPFKEIPREDRNLPQQG---EDGKGFFYNNPKMGRIVKISVYILLHVVVVGYFSYAT--Y
20. D.ficusphila	_A0A1W4UZY5	CNT2	---MSEDSSKGAINNGYELDHKQLEIHDSEEFKELPLENIKEV------SKKGGYFDDNPKVARLVRISLYILLHLVVVGYFSYAT--Y
21. D.busckii	_A0A0M4ED23	CNT2	---MDSKTDAGAINNSYELDHTDLERAKKTDYENDNAQDQVDSAAVMEQE--------QSKLPRFVKIILYVILHGLVVAYFSYAT--Y
22. D.virilis	_B4LP54	CNT2	---MDSKTSTGAINNAYELDHTDVGRSNPESHKTDYQVDYIHDQGPMQNDGGMETQN-YKKLIRWIKIGFQIVLQIGIVGYFSYAT--Y
23. D.navojoa	_A0A484BVE4	CNT2	MDSMGDKDSMGVTNNAYELDHT--DLRRSNPEFNNKTDYQVDYSHDHDLSVDRRETQENSEINKWIKRGLHLILQIAIVGYFVYAT--Y
24. D.grimshawi	_B4J5F4	CNT2	------MDNKGTINNAFELDHTDYEDYTNDDGAIERDRREQE----------------NQKWHRWLKIGLLLILHIAIVVYFSYAT--Y
25. D.mojavensis	_B4KLQ3	CNT2	---MGDKESMGVNNNAYELDHTDLGRSNPEFNKT---DYQVDYSHDHDLSVDRRETQENNEINKWIKRGLHLILQIAIVGYFAYAT--Y
26. S.lebanonensis	_XP_030385480.1	CNT	---MATQTSKGVSNNSYELDQDMDALEPPNPANNKTDFQLANMTEVWSDGKLDAIDQPESKYKRLVRRLLIFLIHVAVVGYFSYAT--Y
27. S.lebanonensis	_XP_030379959.1	CNT	-----------------MAEPVTETETEPEE---------------------------ESPRKKLIKRILLIIFHILFISYFIWCTVVF
28. S.lebanonensis	_XP_030387255.1	CNT	-----------MAKRDNEAQPVLEDETEPEE---------------------------ENKAKRICKLVCLIVFHIVFIAYFIWCTVVY
29. M.domestica	_XP_005188662.1	CNT	-----------------MEDTKSGEINKAFSGDEILENGAETHKTDFQIEDITSNVKEHSKIKRIVMSSLVWLIHIVVGSYFGYAT-HY
30. M.domestica	_XP_011294508.1	CNT	------MIRYKKPKLLWNNELKLLGDNEKKTHV-------------------------ENEAKPL------------------------
31. C.capitata	_XM_004526264.3:148-1959	CNT	-------------METNGQKNTAFEVDDAVENYTAVDTEVQEIDAMESKAEHNRSNT-KRRIKKVARNTLKVGVHFVVGGYFAYAT--Y
32. L.cuprina	_XP_023292620.1	CNT	-------------MEESKEGEINKAYNIDESLENGVENYKTEFQIAEFEDKEKKIKP-KTTLEIWTKRSLLFILHAAVGAYFGYAT--Y

Supplementary Figure 1. Alignment of Cnt1 and Cnt2 protein of *Drosophila* and *Sophophora* subgenus and subgroup. The alignment of the Cnt paralogs shows a marked difference in the first 20 amino acids.

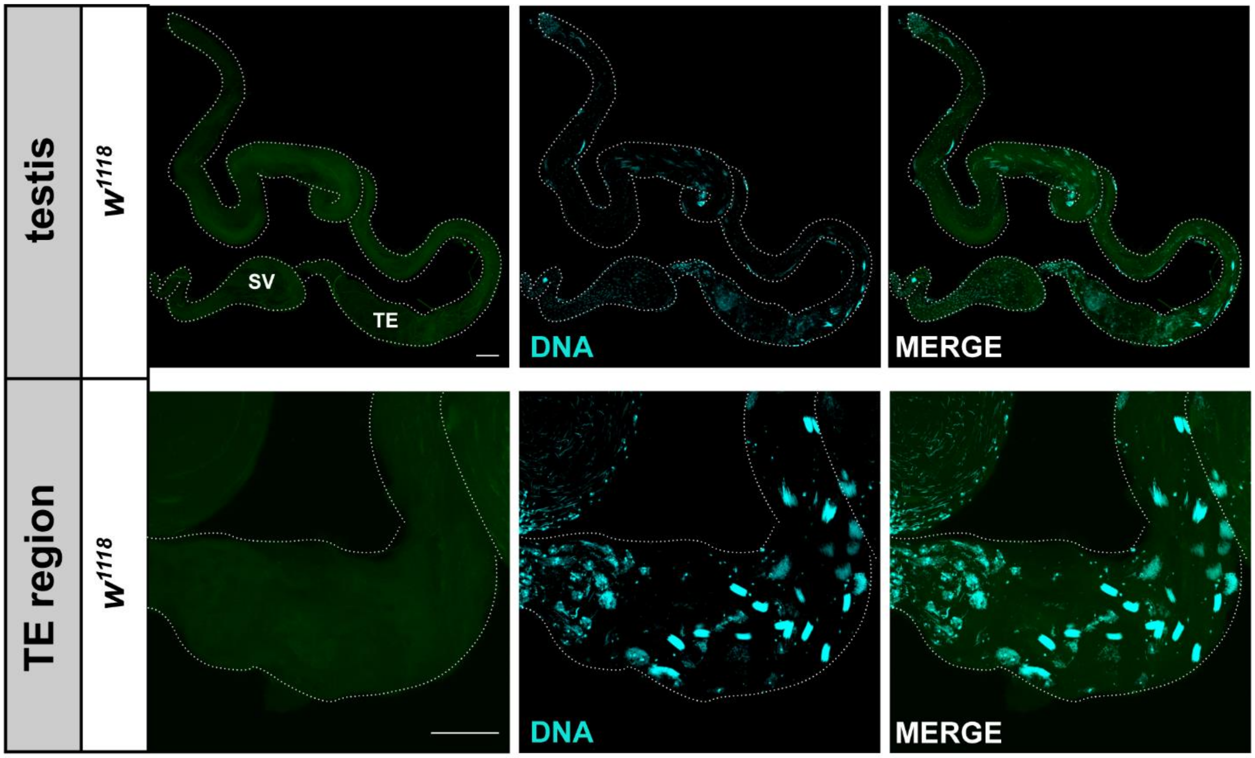

Supplementary Figure 2. Absence of GFP signaling in the w^{1118} fly strain. The figure shows the absence of GFP signal in the testis and its TE region. Scale bar: 40 µm; seminal vesicle (SV), terminal epithelium (TE).

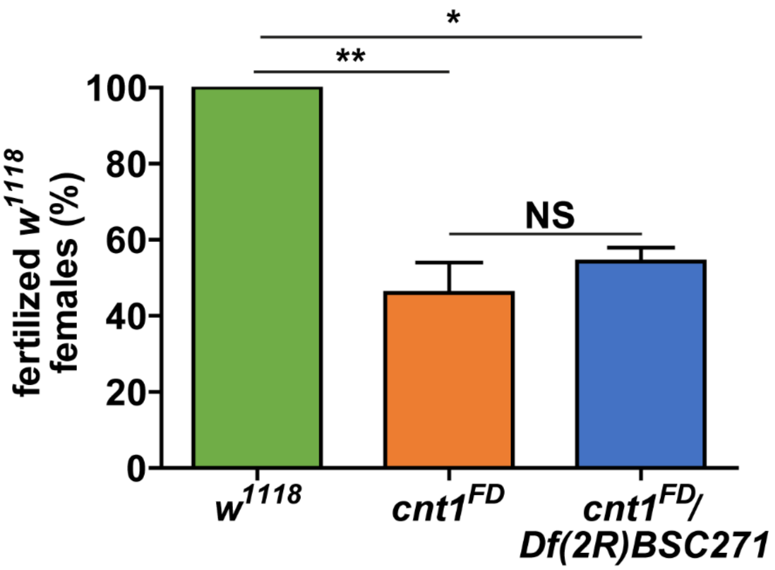

Supplementary Figure 3. The *cnt1* mutation causes partial sterility in *Drosophila* males. Naive mutant males (*cnt1*^{FD}) and control females (*w*^{1118}) were collected. After five to seven days, one male was placed in a vial with 10 females for 24 hours. Subsequently, each female was placed separately in a single vial, and the presence or absence of larvae was recorded. The chart shows the percentage of fertilized females after 24 hours of mating with *cnt1*^{FD} males; n ≥6. Significance was analyzed by Kruskal–Wallis and labeled as follows: *P < 0.05, **P < 0.01, NS > 0.05. Error bars are presented as mean ± SEM.

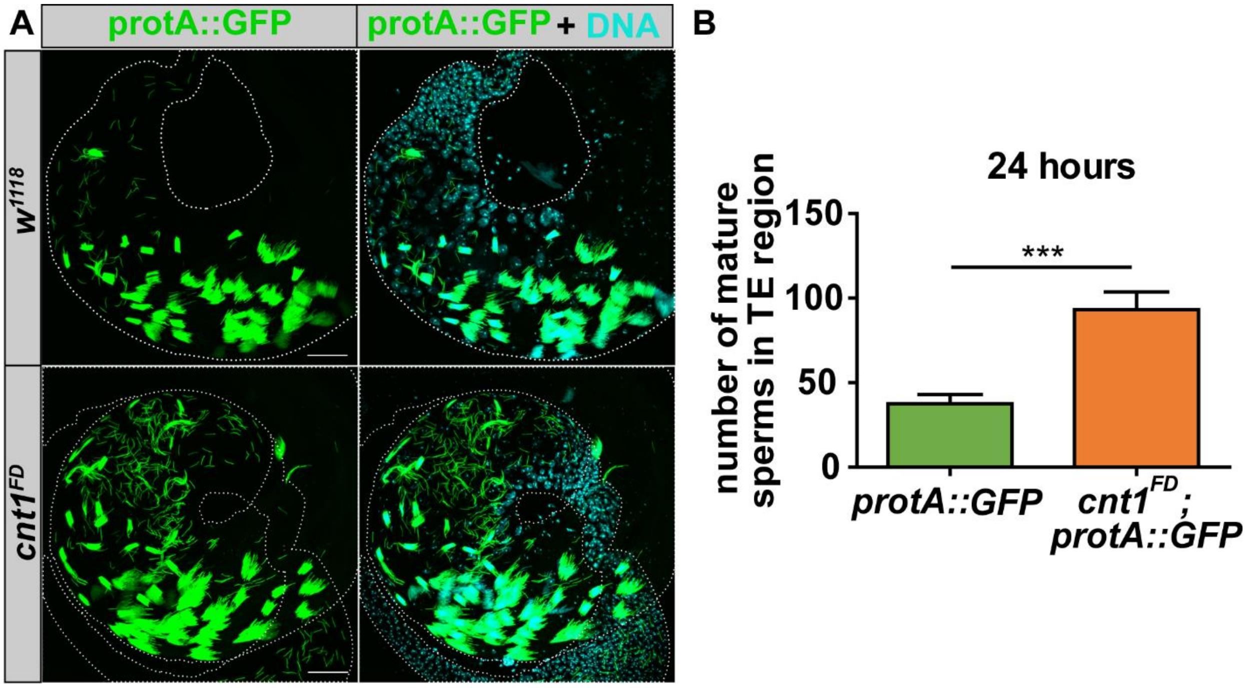

Supplementary Figure 4. The *cnt1* mutation increases the number of spermatid groups at an early age (24 hours old). **(A)** Microscopic observation of spermatids expressing protamine A signal in the TE region of the testis. w^{1118} is used as a control. In w^{1118}, the spermatid groups are localized at the beginning of the TE region whereas the $cnt1^{FD}$ mutants show that the spermatid groups are scattered along the TE region. Protamine A (green), DNA (cyan). Scale bar: 40 µm. **(B)** Number of mature sperms in the terminal epithelium region. Significance was analyzed with a one-tailed Student's t-test and labeled as follows: ***P < 0.001; n = 9. Error bars are shown as mean ± SEM.

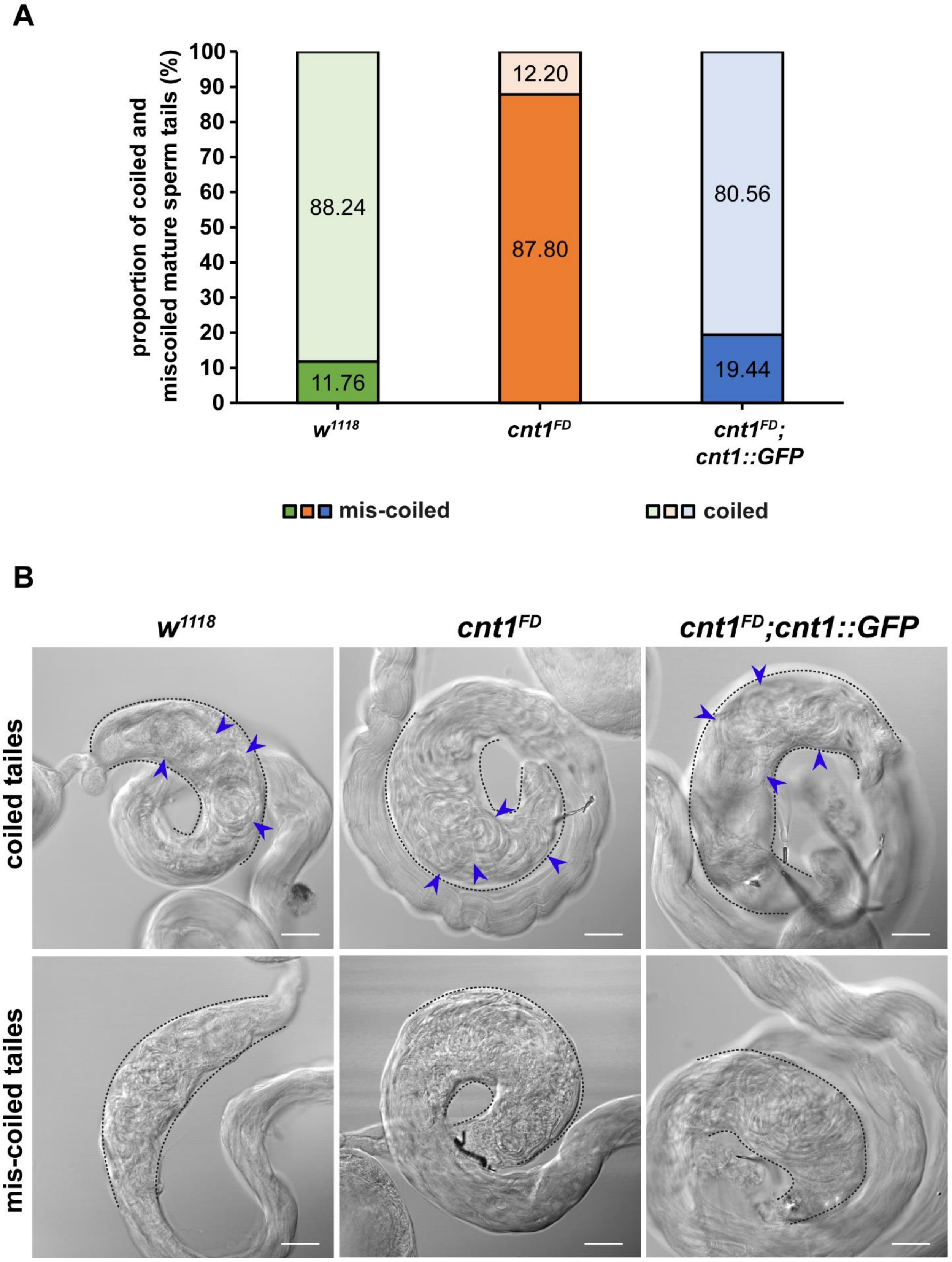
A
proportion of coiled and miscoiled mature sperm tails (%)
100
90
80
70
60
50
40
30
20
10
0
88.24
11.76
12.20
87.80
80.56
19.44
w1118
cnt1FD
cnt1FD; cnt1::GFP
mis-coiled
coiled
B
w1118
cnt1FD
cnt1FD;cnt1::GFP
coiled tailes
mis-coiled tailes

Supplementary Figure 5. The *cnt1* mutation causes a miscoiling of mature sperm groups. **(A)** Proportion of coiled and miscoiled mature sperm tails; n ≥34. **(B)** Microscopic observation of coiled and miscoiled mature sperm tails. The top panel shows an example of coiled tails in the TE region and the down panel shows the miscoiled tails in the TE region of in w^{1118}, $cnt1^{FD}$ and $cnt1^{FD}$;*cnt1::GFP* testis. The blue arrow points to the circular coiled tails. Scale bar: 40 µm.

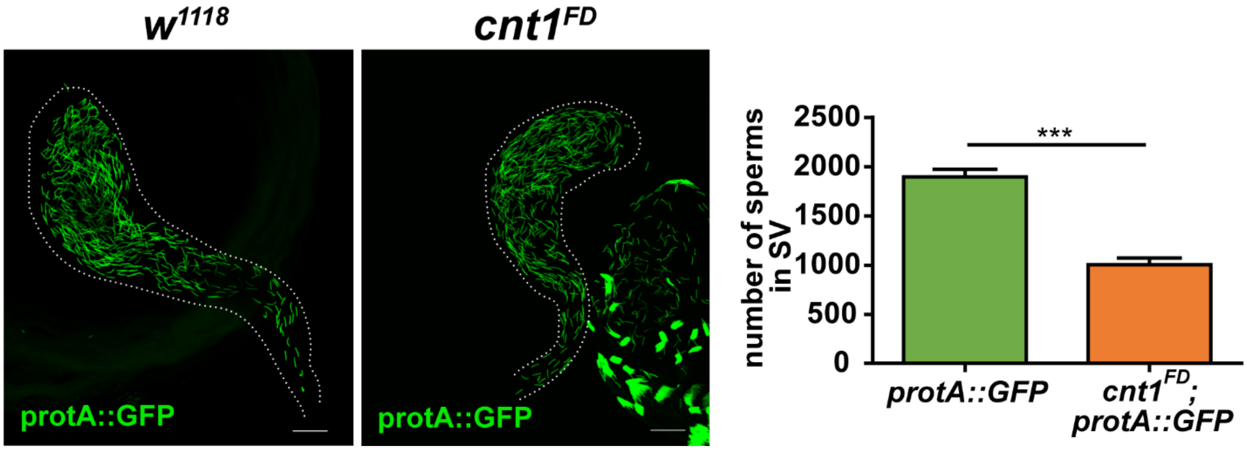

Supplementary Figure 6. *cnt1* mutation causes a defect in the movement of mature sperms toward the SV. **(A)** Microscopic observation of sperms in the SV expressing protamine A signal. Protamine A (green). Scale bar: 40 µm. **(B)** Number of sperms in the SV. Significance was analyzed using one-tailed Student's t-test and labeled as follows: ***P < 0.001; n ≥10. Error bars are shown as mean ± SEM.

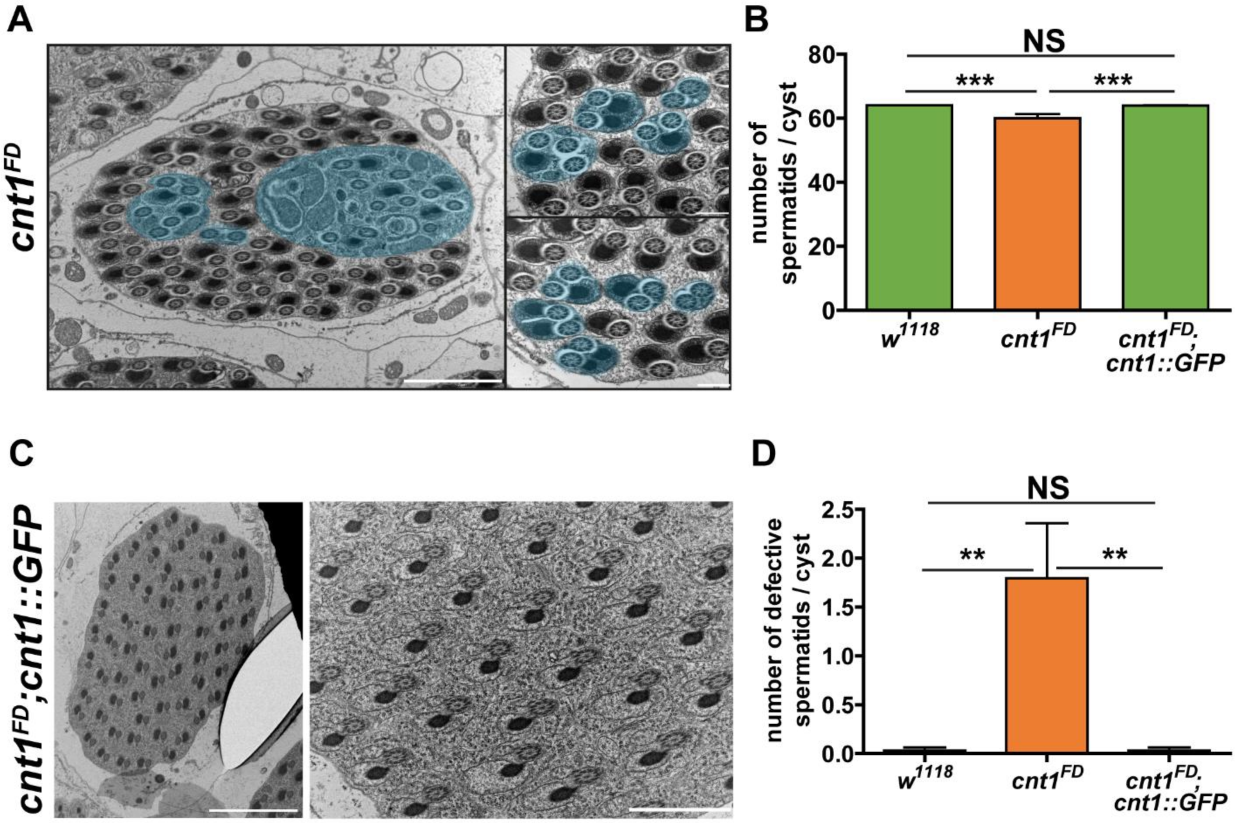

Supplementary Figure 7. *cnt1^{FD}* mutants exhibiting mitochondrial defects in spermatids. **(A)** Spermatid cysts of *cnt1^{FD}* mutants show either a lack of major mitochondria or a mitochondrial fusion of spermatids. Left panel, scale bar: 2 µm. Right panel, scale bar: 500 nm. **(B)** Number of spermatids per cyst. **(C)** Transversal section of *cnt1^{FD};cnt1::GFP* mutant testes showing a cyst of elongating spermatids. Left panel, scale bar: 5 µm. Right panel, scale bar: 2 µm **(D)** Number of defective spermatids per cyst. **(B,D)**; $n \geq 32$. Significance was analyzed by Kruskal–Wallis and labeled as follows: $**P < 0.01$, $***P < 0.001$, $NS > 0.05$. Error bars are presented as mean ± SEM.

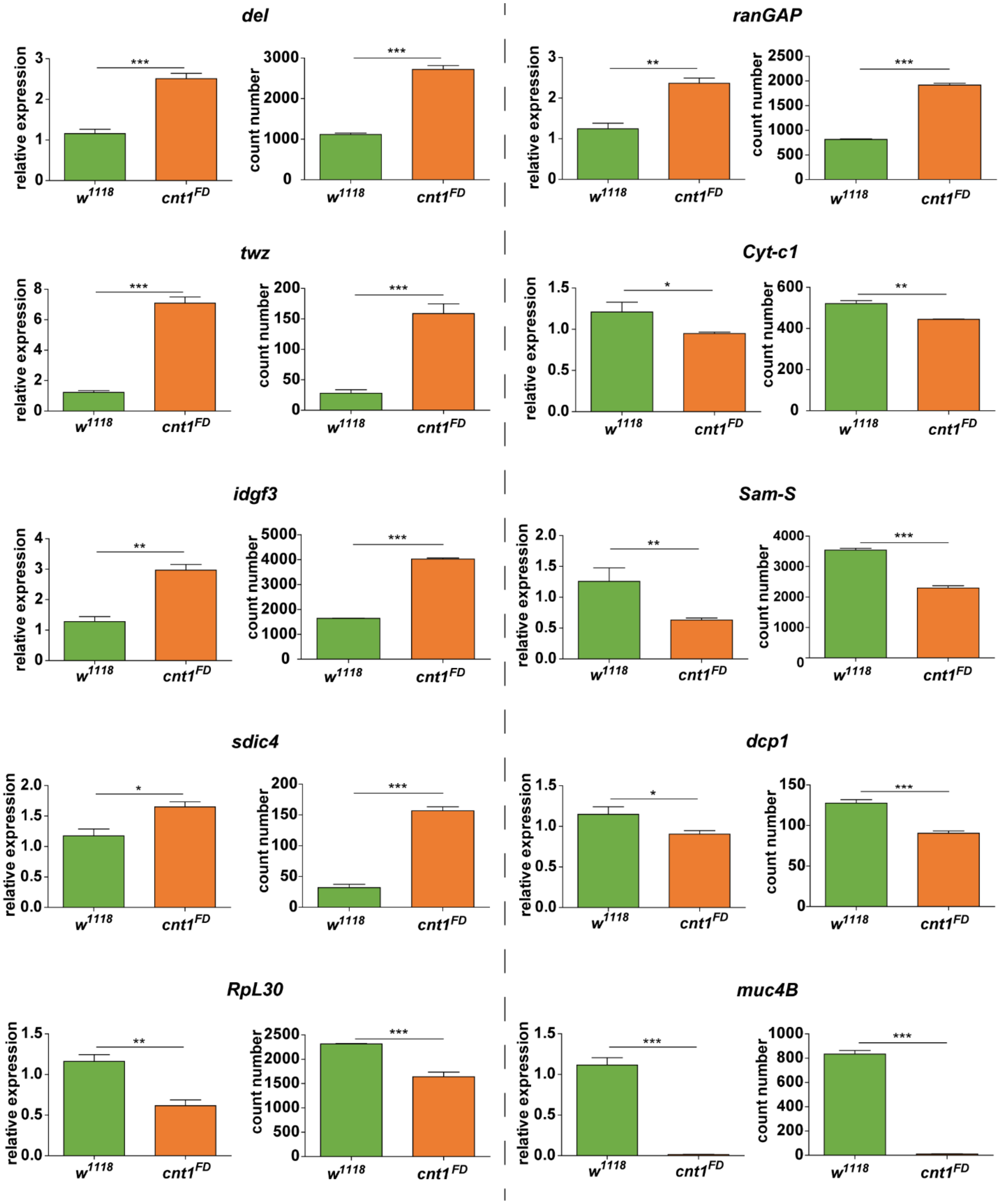

Supplementary Figure 8. Comparison of RNA sequencing and qPCR results for the transcription of 10 representative genes. The figure shows the plot of qPCR data (relative expression)on the left and the plot of RNAseq data (count number) on the right for the following genes: *del* (*deadlock*), *ranGAP* (*Ran GTPase activating protein*), *twz* (*tiwaz*), *Cyt-c1* (*Cytochrome c1*), idgf3 (*imaginal*

disc growth factor 3), *Sam-S* (*S-adenosylmethionine synthetase*), *sdic4* (*sperm-specific dynein intermediate chain 4*), *dcp1* (*death caspase 1*), *RpL30* (*ribosomal protein L30*) and *muc4B* (*mucin 4B*). Expressions were normalized to *αTub84B and act5C* transcripts (ΔΔCT). Significance was analyzed with a one-tailed Student's t-test and labeled as follows: *P < 0.05, **P < 0.01, ***P < 0.001; n = 3. Error bars are shown as mean ± SEM.

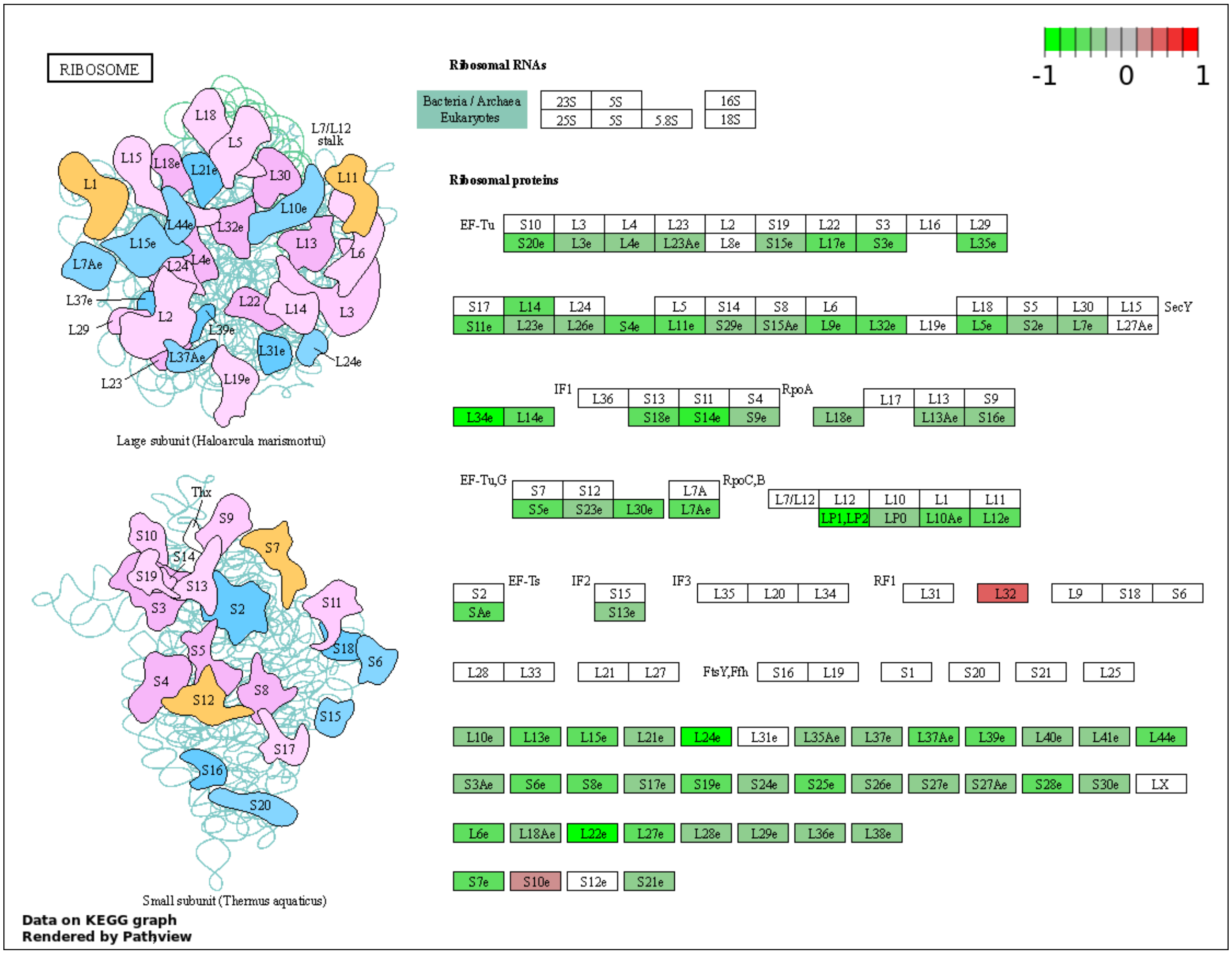

Supplementary Figure 9. KEGG ribosomal pathway: dme03010. Differential expression of ribosomal pathway genes. The figure shows up-regulated genes (red) and down-regulated genes (green).

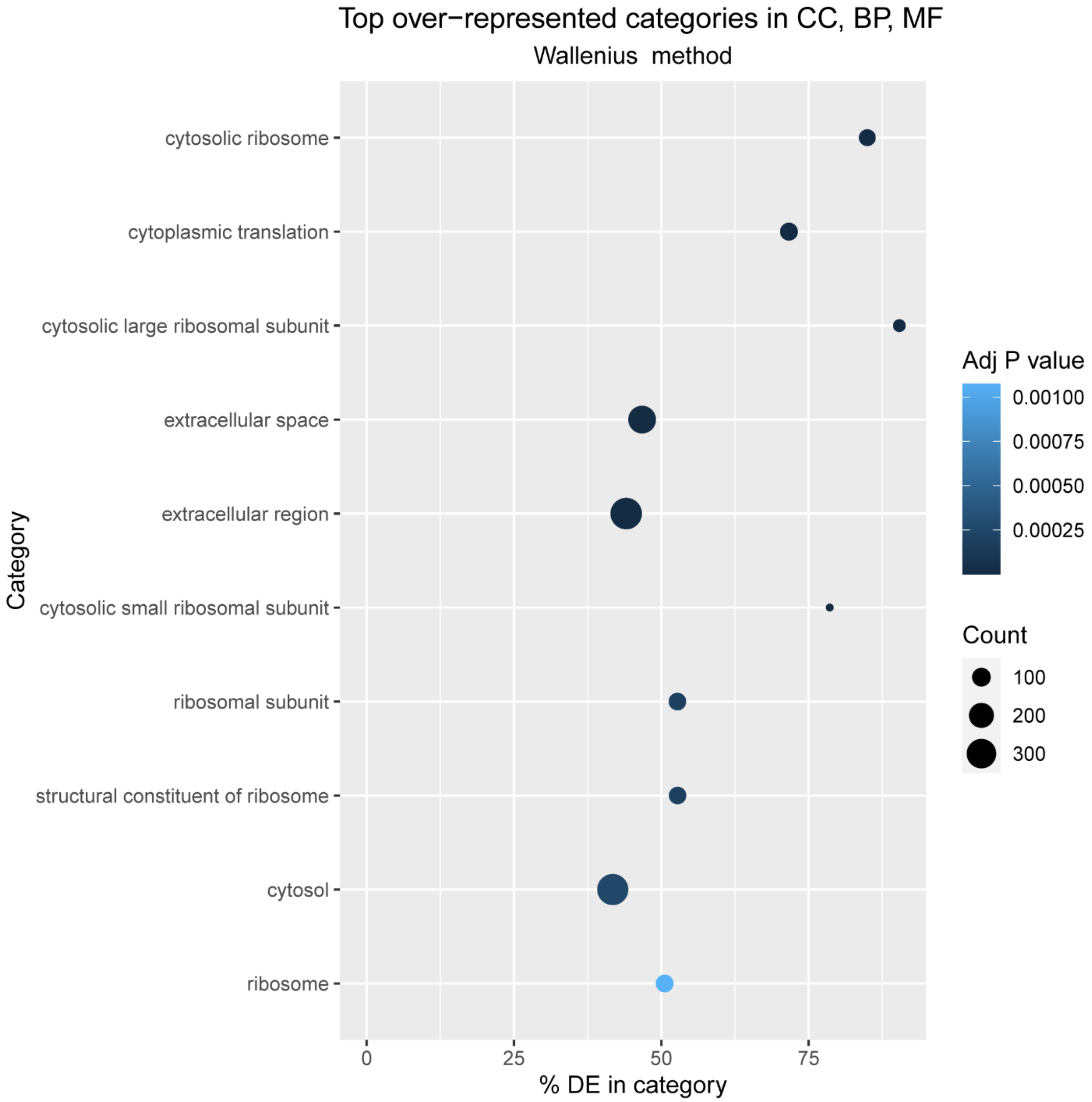

Supplementary Figure 10. A scatter plot of KEGG pathway enrichment statistics. The graph shows the top 10 over-represented GO terms. The x-axis shows the percentage of genes in the category identified as differentially expressed. Molecular Function - molecular activities of gene products (MF), Cellular Component - where gene products are active (CC), Biological Process - pathways and larger processes consisting of the activities of multiple gene products (BP).

Supplementary Table 1. List of used primers.

Supplementary Table 2. Results of the post-hoc tests in tabular form for all pairwise comparative analyses of three groups. Significance was established using parametric test: one-way ANOVA (combined with Tukey post-hoc test), or non-parametric test: Kruskal–Wallis (combined with comparisons of mean ranks of all pairs of groups post-hoc test).

Supplementary Table 3. List of the differentially expressed genes detected in $cnt1^{FD}$ mutants compared with w^{1118}. The genes on the list are the differentially expressed genes based on the adj. P-value < 0.05. The highlighted genes in the Excel file belong to the discovered ribosomal pathway (the red ones are up-regulated, and the green ones are down-regulated). The table lists the genes in the following categories: immune system and inflammation, metabolism, and reproduction.
